# Divergent Innate and Epithelial Functions of the RNA-Binding Protein HuR in Intestinal Inflammation

**DOI:** 10.3389/fimmu.2018.02732

**Published:** 2018-11-23

**Authors:** Eleni Christodoulou-Vafeiadou, Fotis Ioakeimidis, Margarita Andreadou, Giorgos Giagkas, George Stamatakis, Martin Reczko, Martina Samiotaki, Anastasios D. Papanastasiou, Ioannis Karakasiliotis, Dimitris L. Kontoyiannis

**Affiliations:** ^1^Division of Immunology, Biomedical Sciences Research Center Alexander Fleming, Vari, Greece; ^2^Department of Genetics, Development and Molecular Biology, School of Biology, Aristotle University of Thessaloniki, Thessaloniki, Greece

**Keywords:** cytokine regulation, intestinal inflammation and cancer, post-transcriptional regulation, inflammatory bowel disease, animal models of human disease

## Abstract

HuR is an abundant RNA-binding protein acting as a post-transcriptional regulator of many RNAs including mRNAs encoding inflammatory mediators, cytokines, death signalers and cell cycle regulators. In the context of intestinal pathologies, elevated HuR is considered to enhance the stability and the translation of pro-tumorigenic mRNAs providing the rationale for its pharmacological targeting. However, HuR also possesses specific regulatory functions for innate immunity and cytokine mRNA control which can oppose intestinal inflammation and tumor promotion. Here, we aim to identify contexts of intestinal inflammation where the innate immune and the epithelial functions of HuR converge or diverge. To address this, we use a disease-oriented phenotypic approach using mice lacking HuR either in intestinal epithelia or myeloid-derived immune compartments. These mice were compared for their responses to (a) Chemically induced Colitis; (b) Colitis- associated Cancer (CAC); (c) T-cell mediated enterotoxicity; (d) *Citrobacter rodentium*-induced colitis; and (e) TNF-driven inflammatory bowel disease. Convergent functions of epithelial and myeloid HuR included their requirement for suppressing inflammation in chemically induced colitis and their redundancies in chronic TNF-driven IBD and microbiota control. In the other contexts however, their functions diversified. Epithelial HuR was required to protect the epithelial barrier from acute inflammatory or infectious degeneration but also to promote tumor growth. In contrast, myeloid HuR was required to suppress the beneficial inflammation for pathogen clearance and tumor suppression. This cellular dichotomy in HuR's functions was validated further in mice engineered to express ubiquitously higher levels of HuR which displayed diminished pathologic and beneficial inflammatory responses, resistance to epithelial damage yet a heightened susceptibility to CAC. Our study demonstrates that epithelial and myeloid HuR affect different cellular dynamics in the intestine that need to be carefully considered for its pharmacological exploitation and points toward potential windows for harnessing HuR functions in intestinal inflammation.

## Introduction

The effective post-transcriptional control of mRNAs involved in mucosal responses and immunity by specific RNA-binding proteins is emerging as important for the homeostasis of intestinal epithelial barrier (1). In the context of intestinal inflammation, cytokine and other inflammatory mRNAs are actively controlled by degradation or translation regulation within the immune repertoires that act to safeguard the intestinal epithelium and to provide tolerance to commensal microbiota (2, 3). Similarly, the ever-changing life-cycle of resident intestinal epithelial cells and their response to commensal and threatening signals may be affected by selective post-transcriptional programs altering the fate of mRNAs involved in differentiation, proliferation and death (1). The lack of prudent post-transcriptional controls in either mucosal immune cells or constituent epithelia can alter their cross-talk to provide the impetus for uncontrollable inflammation, intestinal degeneration and cancer.

A prototypical system of post-transcriptional control that is seemingly active in immune and epithelial layers of the intestine concerns mRNAs containing U-and AU-rich elements in their untranslated termini. As exemplified by the paradigm of the ARE-containing TNF mRNA (2–5), such mRNAs encode for a variety of factors relevant to the responses of the intestinal mucosa and are targeted by a class of RNA binding proteins (RBPs) such as the decay promoting family of Zfp36 proteins and hnRNPD/AUF1; translational inhibitors like TIA1 and TIAR; several associated factors and non-coding RNAs; and the pleiotropic factor Elavl1/HuR. Although most of these ARE-associated factors have gained attention as post-transcriptional modifiers in immunopathology (2), Elavl1/HuR is a well-studied example of clinical relevance to intestinal diseases. HuR is a ubiquitous and conserved RBP that binds to U- and AU- rich RNA motifs and shuttles between nucleus and cytoplasm via signal-induced interactions (6, 7) with nuclear export/import adaptors. In the nucleus, HuR may act to modify the maturation and/or processing of coding and non-coding RNAs (8). In the cytoplasm, HuR may promote the synthesis of inflammatory mediators and tumor-associated proteins by stabilizing the corresponding mRNAs thus acting as ***a pro-inflammatory and pro-tumorigenic factor*** (9). However, it may also alter ARE-mediated mRNA translation and turnover differentially, through synergies and antagonisms with other RBPs, miRNAs and long non-coding RNAs (10–12). HuR binds to an extensive list of RNAs (13, 14), and as such may appear as non-discriminatory in terms of pathologic and homeostatic functions. However, the expanding list of tissue restricted mouse mutations (15–24) reveal that HuR can have-sometimes unpredictable—tissue and signal restricted functions.

With respect to intestinal epithelia, a pathologic role for HuR is supported by the clinical connection of its –total or cytoplasmic—elevation to intestinal cancers. These elevations correlated: (a) positively to the degree of transformation, malignancy and tumor angiogenesis; and (b) negatively to the overall survival of patients with rectal and colonic tumor (25–32). A multitude of cellular studies connected HuR to the stabilization of mRNAs promoting cancer traits like tumor cell proliferation, survival, tumor angiogenesis, and metastasis (33, 34). Most experimental data stem from such cellular studies or from the xenotransplantation of tumor epithelia, and point toward the regulation of cell cycle and proliferation as *the* major function regulated by HuR in intestinal epithelia (29). A set of genetic studies supported this notion; when HuR was deleted inducibly post-birth in intestinal epithelia, its loss hampered epithelial regeneration under several conditions whereas models of colitis associated cancer (CAC) and APC driven cancers showed signs of remission (16). A pathologic profile of elevated total HuR has been detected in histological samples from active IBD-namely Crohn's disease and Ulcerative Colitis. Collectively, these observations rendered HuR as target of clinical relevance in intestinal disease and colon cancer; and culminated the search for specific pharmacological modulators inhibiting HuR's translocation or binding (35).

However, disparate data did point toward a differential role for HuR in both the intestinal epithelium and mucosal immunity. When HuR is deleted acutely post-birth, its loss leads either to barrier degeneration and progenitor loss (if deletion is systemic) or villus shortening (if deletion is IEC-restricted) connecting to developmental changes in cell survival and death (15, 16). However, when deleted earlier and continuously in IECs, its loss induces a partial shortening of jejunum villi but does not affect intestinal ontogeny and barrier function (18); however and depending on the challenge, the latter group of mice reveal problems either in regeneration or cadherin-mediated junctions (18, 36). These studies provide support for HuR functions in IEC ontogeny, survival, and barrier integrity. In the context of IBD, and although HuR appears elevated in inflamed epithelia, its expression in transitory dysplastic epithelia connecting IBD to CAC seems to reduce to normal levels (37). With regard to its function in inflammatory cells, HuR's sole function as an RNA activator has been revisited, primarily because genetic studies on innate immune effector cells did not fully support this notion. In mice rendered deficient for HuR in myeloid cells and the immune derivatives, inflammation was not suppressed but rather enhanced to a pathologic extent (20, 38). With respect to mucosal responses, these mice displayed an exacerbated response to the model of chemical colitis and –most profoundly- to CAC (20). The opposite experiment was even more revealing with elevated macrophage HuR suppressing pro-inflammatory reactions including chemical colitis and CAC (17, 20).

The consideration of HuR inhibition as a therapeutic strategy against intestinal inflammation and cancer was most profoundly challenged during the pre-clinical testing of one of its pharmacological inhibitors (37). In models of familial CRC, HuR inhibition appeared effective in suppressing tumor growth and progression. In the context of inflammatory CAC, HuR inhibition not only failed but also exacerbated tumor progression.

As such the current data as to whether HuR plays a pathologic or protective role in intestinal inflammation appear ambiguous possibly due to divergent cell type specific effects. Here we focus on two prototypical subsets located in the intestinal mucosa, involved in inflammatory disease—myeloid-derived immune cells vs. intestinal epithelia- and dissect the functions of HuR in several models of pathologic and beneficial inflammation. Our data reveal that the cell-restricted functions of HuR drive divergent, non-overlapping and context-dependent inflammatory responses in the intestinal mucosa, altering the clinical outcome of intestinal disease that need to be considered for clinical intervention.

## Materials and methods

### Mice and study approvals

*Elavl1*^*fl*/*fl*^, *VillinCre*^+^, *LysozymeCre*^+^, *LysozymeCre*^+^*HuR*^*fl*/*fl*^, and *Tnf*^Δ*ARE*/+^ mice have been previously described (4, 17, 20, 39). To generate *VillinCre*^+^*Elavl1*^*fl*/*fl*^ mice, *Elavl1*^*fl*/*fl*^ mice were crossed with *VillinCre*^+^. For *TgATFHuR*^+^ mice the complete cDNA sequence for human HuR was obtained from the IMAGE clone number 2901220 (GenBank accession number BC003376) and was subcloned in-frame to an AviTEVFLAG (ATF) sequence for the expression of an N-terminally tagged form. Fusion was verified by sequencing. Subsequently, the ATF-HuR cDNA was used for the generation of the transgenic construct. The device was removed from the carrier vector via the digestion of flanking PmeI sites. For the production of transgenic mice, fertilized C57Bl/6J zygotes were coinjected with the transgenic device procedures employed by the INFRAFRONTIER-GR/Trangenesis Unit of BSRC “Al. Fleming” (http://www.infrafrontier.gr/). To identify and maintain transgenic founder mice, tail DNA was used for Southern blot hybridizations and PCR with specific probes and primers to detect the transgene. Five founder lines were identified; line Tg6105 is the one employed in this study. All mouse lines were maintained in a C57BL/6J background and in the animal facilities of the BSRC “Alexander Fleming” under specific-pathogen free conditions. All experiments were performed with mice aged between 8 and 16 weeks, with a minimum of 3 mice per genotype and a maximum of 30, according to the experiment, as indicated in the figure legends. Littermates were used as controls. Animal experiments were approved by the Prefecture of Attica (licenses #5995/2012, 4371-4376/2014, #6198/2017, #3547/2018, #2824/2018) in accordance to national legislation and the European Union Directive 63/2010.

### DSS colitis and CAC

Mice 6–8 week old were fed *ad libitum* for up to 2 cycles with water containing 1.5–2% (wt/vol) DSS (MW 40,000 kDa; MP Biomedicals Inc.) for 6 days, followed by intervals of 15 days on regular water. For induction of CAC, 6–8-week-old mice were injected i.p. with 20 mg/kg mouse DMH (SIGMA-Aldrich). After 5 days, 1.5–2% (wt/vol) DSS was provided in drinking water for 6 days, followed by 15 days of regular water. This cycle was repeated twice. During the course of the experiment of acute inflammation or tumorigenesis, mice were monitored daily for body weight, diarrhea, and rectal bleeding. Values were used for calculation of DAI (40, 41). Mice were sacrificed at indicated time points or at the end of the protocol (15 weeks) for the isolation of colonic tissue. Tumor sizes were measured using an electronic Vernier caliper.

### Colon explant cultures

Colon explant cultures were performed as previously described (20). Briefly, whole colons were opened longitudinally, washed with PBS supplemented with 20 mg/ml gentamycin to remove residual intestinal bacteria and cut in 1.5-cm pieces into a 48-well plate containing 500 μl RPMI-1640 per well. Tissues were incubated at 37°C, 5% CO_2_ for 24 h, and supernatants were collected for cytokine/chemokine ELISA measurements.

### Immunoblotting and ELISAs

For Western blots, lysates were analyzed on SDS-polyacrylamide gels (7–14%), along with protein molecular weight markers (SM0431; Nippon MWP03; Fermentas, Thermo Scientific) and blotted onto nitrocellulose membrane (GE Healthcare). After blocking with 5% milk or 4% BSA in TBS-Tween 20 buffer, membranes were incubated with primary and HRP-conjugated secondary antibodies; signals were visualized by enhanced chemiluminescence (ECL; GE Healthcare) using films or a ChemiDocTMXRS+ System with Image LabTM software. Antibody used: HuR (Santa Cruz, sc-5261). Supernatants were analyzed via specific ELISAs (Peprotech; eBioscience) or Cytometric Bead Arrays (BD Biosciences).

### Histology and immunohistochemistry

Dissected intestines were mounted onto a solid surface and fixed in formalin 10% (pH = 6.9–7.1) O/N at 4°C before processing for paraffin embedding. At least 2 serial sections of 5 μm were stained with: hematoxylin and eosin (H&E) for general histology. Periodic Acid Schiff and Nuclear Fast Red stains were carried out using standard protocols. For Immunohistochemistry of paraffin embedded tissues sections were deparaffinized, hydrated, and treated with boiling Citrate buffer pH 6.0 under microwaves for 20 min. Sections were blocked in diluent (0.1% gelatin, 0.5% Triton-X, 0.05% Tween-20 in TBS) supplemented with 1%BSA, 3% FBS, and 3% H2O2 for 1 h. Primary (HuR 3A2, SantaCruz, sc-5261; Lysozyme, DAKO Cytomation, A0099; Ki67, Abcam, ab15580 (*TgATFHuR*^+^ characterization) or Thermo, MA5-14520 (*C. rodentium* experiment) and HRP-conjugated secondary antibodies (Southern Biotech) were incubated in diluent for 1–24 h. Visualization was performed with DAB (Vector) and counterstained with hematoxylin; Photomicrographs were acquired using a Nikon ECLIPSE E200 microscope equipped with a Nikon Digital Sight DS-5M digital camera.

### T cell mediated enteropathy

Mice received a single i.p. injection of 50 μg hamster anti-CD3 antibody (2C11; LEAF grade-Biolegend) in saline and were fasted 16 h before their sacrifice. Mice were sacrificed at different time points for histology and luminal exudate collection. For the latter, the whole intestine was removed, the small intestine was cut in two halves and its luminal exudate was collected with two flushes of 5 ml PBS, 5% FBS for each half. Fecal pellets were gently removed from the colon using forceps before collecting the luminal exudate in 5 ml of the same buffer. For the flow cytometric detection of cell death, cells were stained with PI or 7AAD (Sigma) and Annexin-V (eBioscience) and were analyzed with a FACSCanto II flow cytometer. Analysis of luminal cells was performed as in Piguet et al. (42).

### Citrobacter rodentium infections

*C. rodentium* strain DBS100 (ATCC 51459) was cultured to exponential phase (1 < OD_600_ < 1.4) in Luria-Bertani Broth overnight at 37°C with shaking at 200 rpm. Culture was spun 10 min at 3,000 g at 4°C the pellet was washed twice in ice-cold PBS and cells were resuspended in ice-cold PBS. Eight to twelve weeks old mice received an oral gavage of 2 × 10^9^ CFUs in 200 μl suspension in PBS after 8 h of fasting. To reduce mouse-to-mouse transmission, mice were caged single or in duplet. For CFU determination in feces, 20–60 mg of fecal matter was collected, weighed and homogenized in 1 ml PBS, serial dilutions were prepared in PBS in 96-well plates and single drops of 10 μl from each dilution were plated in duplicate on McConkey-agar plates before overnight incubation at 37°C. CFUs were counted from the same dilution for every sample and were normalized with respective fecal weight. *C. rodentium* colonies were identified as pink with a narrow white trim. Experiments with *C. rodentium* were carried out in the Biosafaty Level 2 facility of “Alexander Fleming.”

### Microbiome analysis

Fecal samples were collected fresh, weighed, and homogenized in 1 ml PBS at room temperature before transfer to −80°C until use. Samples were thawed and centrifuged at 100 g for 3 min. The supernatant was mixed 1:1 with 2x lysis buffer (8% SDS, 0.2 M DTT, 0.2 M Tris-HCl pH 7.5) and subjected to heating at 95°C for 5 min and pulsed probed sonication 30 s × 2 with 45 s interval. The sample was finally cleared from insoluble material by centrifugation at 14,000 g for 20 min. The protein extract was subjected to trypsin digestion using the sp3 procedure (single pot protocol) (43). The resulting peptides mix were analyzed with LC-MS/MS using a 4 h gradient as described in Elkouris et al. (44). Methods for data processing is provided in Supplementary Material.

### Histopathological scoring of DSS colitis and CAC, T-cell mediated enteropathy, and *C. rodentium* infection

The histological scoring system of DSS-induced colitis and CAC was described in Yiakouvaki et al. (20). For DSS-colitis it included severity and extent of inflammation (ranging from 0 to 3), crypt damage (from 0 to 4), and percentage of organ affected (from 0 to 4). For *C. rodentium* colitis, inflammation scoring system was as for DSS and crypt damage was scored in a scale of 0–4 (none, rare, occasional, frequent, extensive) for frequency of observed damaged crypts. For T-cell mediated enteropathy, the histological score was calculated as for DSS-colitis. For colonic crypt length measurements, using the 10x objective lens the whole distal colon was divided in optic fields, photos were taken from every field that had at least one well-oriented crypt (visible from base to apical opening) and the length of at least 12 well-oriented crypts was measured, using the ImageJ image processing software, and was averaged for each mouse. Same strategy was followed for the measurement of small intestinal villi and crypts at the ileum. Well-oriented villi with their associated crypts were measured. All histological assessments were performed in a blinded fashion.

### Crypt proliferation

Paraffin sections were processed for staining with anti-Ki67 antibody. Labeling index was defined as the frequency of Ki67^+^ cells in each crypt and/or villus compartment for each position, from position 1 (base of crypt) to position 40. Fifteen well-oriented crypts were examined for each mouse and positive counts for each position were summed and expressed as percentage of the total cells counted.

### RNA extraction and qRT-PCR

Tissues were dissected, transferred in 0.5 ml Tri Reagent (MRC, USA), snap frozen in liquid nitrogen and stored at −80°C until RNA extraction. Total RNA was extracted after homogenization using an Ultra-Turrax homogenizer (IKA, Germany) according to manufacturer's instructions. RNA integrity was verified with agarose gel electrophoresis and concentration was measured with a Nano-Drop device. One to two μg of total RNA was subjected to DNase treatment (RQ1 RNase-Free DNase, Promega, Madison, WI, USA) and reverse transcribed with M-MLV Reverse Transcriptase (Promega, Madison, WI, USA) using an oligo(dT) primer. qPCR was performed using EvaGreen SsoFast mix (Bio-Rad, Hercules, CA, USA) on a RotorGene 6,000 machine (Corbett Research, Qiagen, Venlo, Netherlands). Expression was normalized to β2-microglobulin. The relative mRNA expression in the test samples was calculated as the difference from the control values that were assigned an arbitrary expression value of 1, using Bio-Rad RelQuant (Bio-Rad). Primers used are provided in Supplementary Material.

### Intestinal epithelial cell and immune cell isolation

For IECs, intestines were opened longitudinally, washed with PBS and cut into 1.5 cm pieces that were washed with inversion in tubes containing HBSS with 2% FBS and 1 mM HEPES. Pieces were transferred to 20 ml HBSS supplemented with 2% FBS, 2 mM HEPES, 5 mM EDTA, and 1 mM DTT and placed at a rotary and at 37°C for 1 h. After setting for 15 min, suspensions were filtered through sterile gauze and centrifuged at 1,200 rpm for 5 min. Epithelial cells were isolated via centrifugation through 25–40% discontinuous Percoll gradient at 600 g for 15 min. The isolated fractions were then used for lysis and immunoblotting. The isolation of bone marrow derived macrophages (BMDMs) and non-adherent splenocytes were performed as in Papadaki et al. and Yiakouvaki et al. (19, 20). Briefly, for BMDMs, bone marrow was isolated from tibias and femurs by flushing, treated with Gey's solution to remove erythrocytes and cultured for 8 days in RPMI (5% FBS) supplemented with antibiotics, glutamine and supernatant from L929 cell culture. These were used for immunoblots or were stimulated with LPS (100 ng/ml) for 24 h for measurement of cytokines. For non-adherent splenocytes, cells were collected using standard procedures and were stimulated with PMA/ionomycin (10/500 ng/ml) for 24 h.

### Ribonucleoprotein immunoprecipitation (R-IP)

Four colonic tumors, of similar size, per mouse from day 60 DMH/DSS treated mice were pooled and snap frozen to create a sample. Three samples per genotype were used. R-IP experiments were performed as described previously (45). Briefly, cells were lysed in buffer containing 100 mM KCl, 25 mM EDTA, 5 mM MgCl_2_, 10 mM HEPES, 0.5% Nonidet P40, 2 mM DTT, 0.2% vanadyl ribonucleoside complex (Invitrogen) and 100 U/ml RNAse OUT (Invitrogen). Antibody coated beads (agarose or protein A-sepharose) were washed and maintained in 750 μl of NT-2 buffer (50 mM Tris, pH 7.4, 150 mM NaCl, 1 mM MgCl2, and 0.05% Nonidet P40). For IP, 200 μl of lysate were loaded onto the beads and incubated for 4 h on a rotary at 4°C. Subsequently, beads were washed five times in NT-2 buffer plus two times in NT-2+1M urea and finally resuspended in NT-2. Ten microliters of samples were removed for immunoblot verification and the remaining were used for RNA isolation via proteinase K treatment, phenol/chloroform extraction and precipitation. Antibodies included unmodified anti-FLAG (M2; Sigma) and mouse immunoglobulin antibodies (Santa Cruz and R&D). Methods for Microarray (Chip) Profile Analysis is provided in Supplementary Material.

### Statistical analysis

All data were analyzed with Graphpad Prism 6.01 (Graphpad Software). Appropriate statistical tests for each experiment are indicated within the figure legends except for R-IP-Chip data analysis where statistical analysis is provided in Supplementary Material.

## Results

### A similar role for intestinal and myeloid HuR in the control of chemically induced colitis but a differential role in CAC

To dissect the functions of HuR in intestinal inflammation, we compared the effects of its deletion in intestinal epithelium to those incurred by its deletion in myeloid-derived immune compartments. This entailed the comparative analyses of mice harboring a loxP flanked *Elavl1*^*fl*/*fl*^ allele (46) rendered as inactive either (a) in intestinal epithelial cells by means of a *Cre* gene driven by a Villin promoter (*VillinCre*^+^*Elavl1*^*fl*/*fl*^ named hereafter as IEC-HuRko mice); or (b) in myeloid-derived cells by means of a *Cre* driven by a Lysozyme M promoter (*LysMCre*^+^*Elavl*^*fl*/*fl*^ named hereafter as M-HuRko mice;) (20).

**Figure 1:**
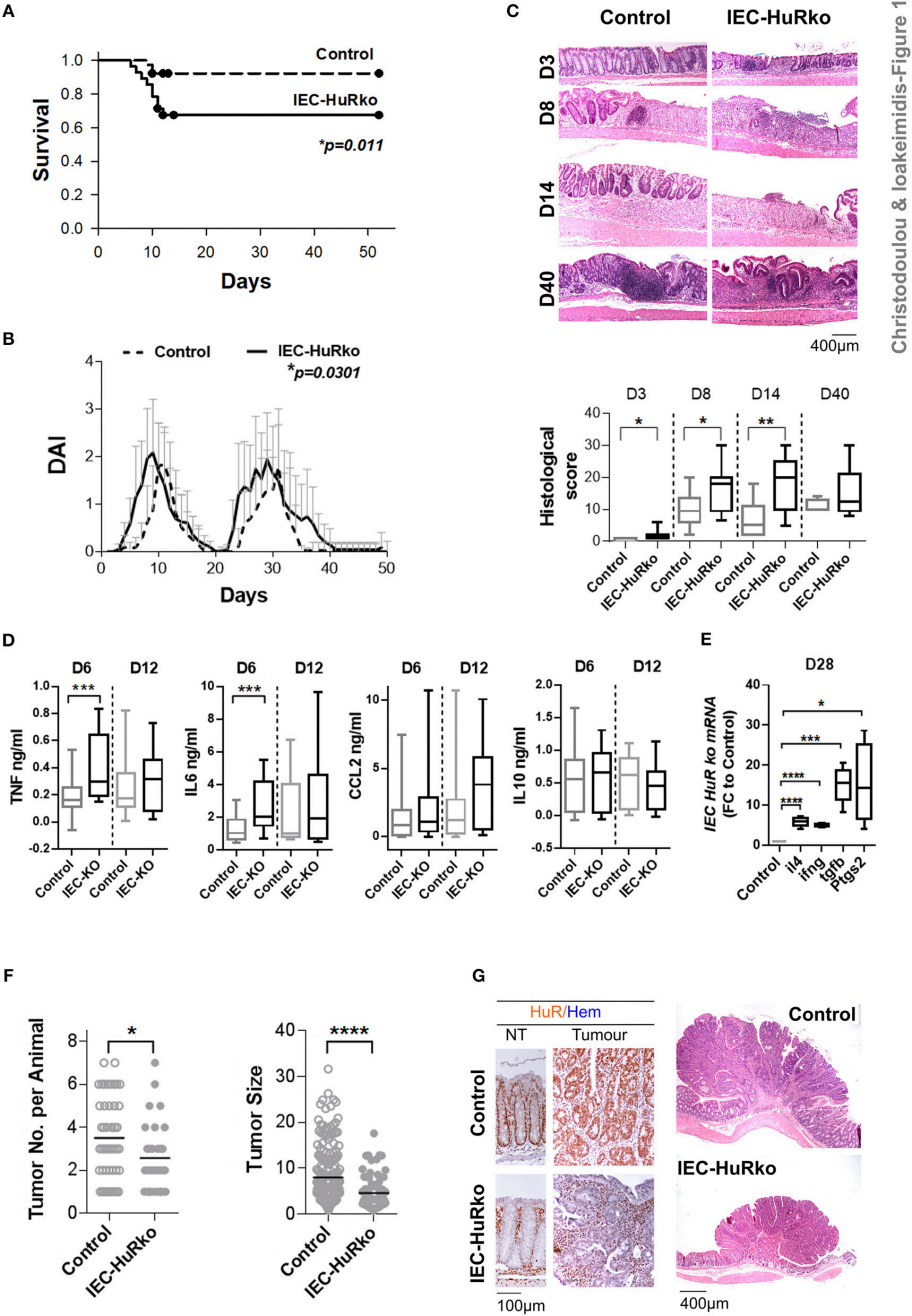
Loss of IEC HuR exacerbates DSS-colitis but attenuates CAC. **(A)** Kaplan-Meier survival analysis following treatment with two rounds of DSS (Days 0 and 20). *n* = 30/group. *p*-value denotes result of Log-rank test. **(B)** Macroscopic Disease Activity Index (DAI) of the same experimental group as in **(A)**. Line graphs depict mean values (± SD). *p*-value denotes result from Kolmogorov-Smirnov test. *n* = 20–30 live mice/group. **(C)** Representative histology (H&E) and histological scores from control and IEC-HuRko mice treated with DSS indicating differences in acute inflammation and ulceration (Days 3–8), epithelial restitution (Day 14) and chronic inflammation (Day 40). *n* = 8–12 mice/group. **(D)** ELISA quantification of inflammatory mediators in colon cultures isolated from DSS-treated mice at the indicated time points. Horizontal lines of box plots represent medians. *n* = 3 cultures/group/time point. Student unpaired *t*-test. **(E)** qPCR detection of mRNAs from DSS-treated colons of IEC-HuRko mice on day 28 after treatment expressed as fold change (FC) relative to control mice. *il4, interleukin 4, ifn*γ, *interferon gamma*, tgfβ, *transforming growth factor beta, Ptgs2, Prostaglandin-Endoperoxide Synthase 2. n* = 5/group. Student unpaired *t*-test. **(F)** Graphs depicting tumor number (left) and tumor size (right) in control and IEC-HuRko mice 60 days after challenge with DMH/DSS. Dots represent individual measurements and horizontal lines represent means. Student unpaired *t*-test. **(G)** (Left panel) Immunohistochemical detection of HuR in colon tumors and adjacent normal tissue (NT). (Right panel) Representative histology (H&E) of colonic tumors in control and IEC-HuRko mice. All box plots show range and median. In all graphs, *,**,***, ****, denote *p*-values < 0.05, 0.01, 0.001, and 0.0001 respectively. Please compare the response of IEC-HuRko mice to the one presented in Figure S1 for M-HuRko mice.

First we analyzed the responses of IEC-HuRko mice to a combined mouse model of colitis and CAC, compared to those we previously published for M-HuRko mice conducted under the same experimental conditions. In this model, disease is induced via a first challenge with a pro-carcinogen (dimethylhydrazine-DMH), and then fed with an inflammatory agent (dextran sodium sulfate-DSS) in repetitive 6-day cycles intermitted by water cycles (47). In control mice, the initial DSS/water cycle led to colitis between days 4–10, which regressed by day 12 (Figures 1A–C). A second DSS/water cycle from day 20 led to a more chronic response between days 23 and 35. IEC-HuRko mice appeared more vulnerable to this challenge; during the initial DSS cycle they manifested partial mortality and an earlier macroscopic disease onset relative to controls (Figures 1A–C). The fast clinical activity of IEC-HuRko mice correlated with aggressive histological features, including the rapid recruitment of mucosal infiltrates and extensive epithelial ulceration even on the 3rd day of the challenge and a delay in epithelial regeneration/restitution (Figure 1C). Similarly, during the chronic phase, IEC-HuRko mice showed persistent disease activity, inflammation and extensive tissue damage (Figures 1B,C). Analysis of afflicted colons revealed the heightened accumulation of selective inflammatory mediators in IEC-HuRko mice, namely TNF and IL-6 at the acute phase (day 6), which dropped to control levels during remission (day 12) (Figure 1D). Interestingly, other known inflammatory HuR targets like CCL2 and IL10 remained unaltered. mRNAs of inflammatory lymphokines and cytokine effectors, such as *ifn*γ, and of *il4, Ptgs-2*/*Cox2*, and *Tgf*β known to be associated with chronic responses (day 28) were significantly upregulated in the colons of IEC-HuRko mice compared to controls, reflecting an exacerbated chronic inflammatory response (Figure 1E). The clinical response of the IEC-HuRko mice to DSS-driven intestinal inflammation phenocopied in part that previously reported for M-HuRko mice [Figure S1 and (20)]. Differences included the much faster degeneration of the colonic epithelial layer during the third day of the protocol, associated with signs of early mortality in the IEC-HuRko mice, as opposed to the heightened and prolonged responses in M-HuRko mice (Figures 1B,C and Figure S1). Qualitatively however, our data indicate that epithelial and myeloid HuR share the same end effect toward the control of inflammation and the protection of the epithelial barrier from degeneration during the DSS challenge.

In contrast, the responses of the cell restricted, HuR mutant mice to the tumor phases of the protocol diverged. In the DMH/DSS model, inflammation enhances dysplasia toward CAC after the 40th day of challenge. As published, the increased inflammatory response of M-HuRko mice to the DSS/AOM challenge correlated with increased tumor formation, growth and progression [Figure S1, and (20)]. Although the increased inflammation in IEC-HuRko colons would point toward a respective progression in tumorigenesis, we found a decrease in the number of tumors per mouse and a profound decrease in the size of these HuR-deficient tumors (Figures 1F,G). Taken together, these data show that in the context of DSS-colitis, and although epithelial and myeloid HuR have a protective role in suppressing inflammation, they have divergent and exclusive roles in CAC signifying their contributions in different inflammatory processes.

A possible explanation for the different tumorigenic response could be that the loss of myeloid HuR elicits different chronic inflammatory responses than those driven by the loss of HuR in IECs. In contrast to the first acute challenge elicited by DSS which drives mainly innate immune responses, the chronic and tumor phases of the model require the additional contribution of the adaptive arm of immunity. To reciprocate such a complex chronic response, we switched to a genetic model of Crohn's like chronic inflammatory bowel disease driven by chronic TNF synthesis, namely the *Tnf*^Δ*ARE*/+^ mouse (4). Due to an induced deletion in the 3′ARE elements of the murine *Tnf* mRNA, the biosynthesis and functioning of TNF in these mice can no longer be modulated at the post-transcriptional level by ARE-binding proteins (including HuR). The uncontrolled production of TNF supports the spontaneous development of inflammatory ileitis in *Tnf*^Δ*ARE*/+^ mice, which becomes histologically evident by the age of 2 months and it progresses continually leading to transmural infiltrates and loss of mucosal architecture by the age of 6 months. The progression of chronic IBD in these mice requires an interplay between innate, adaptive and tissue resident cells (4, 48–50) and as such it approximates the complexities of chronic intestinal inflammation which could be affected differently by the loss of myeloid or IEC HuR. Strikingly however, disease initiation and progression in IEC-HuRko *Tnf*^Δ*ARE*/+^ mice and M-HuRko *Tnf*^Δ*ARE*/+^ mice appeared similar to that in control *Tnf*^Δ*ARE*/+^ mice (Figure S2). We did note however a small delay in the damaging effects in transmural inflammation in M-HuRko mice past the 4th month of age. Still, histological hallmarks of IBD were comparable in all mouse cohorts suggesting that chronic inflammatory processes were not affected by the cell-restricted loss of HuR.

### An exclusive role for intestinal epithelial HuR in the death response elicited via acute inflammatory signals

**Figure 2:**
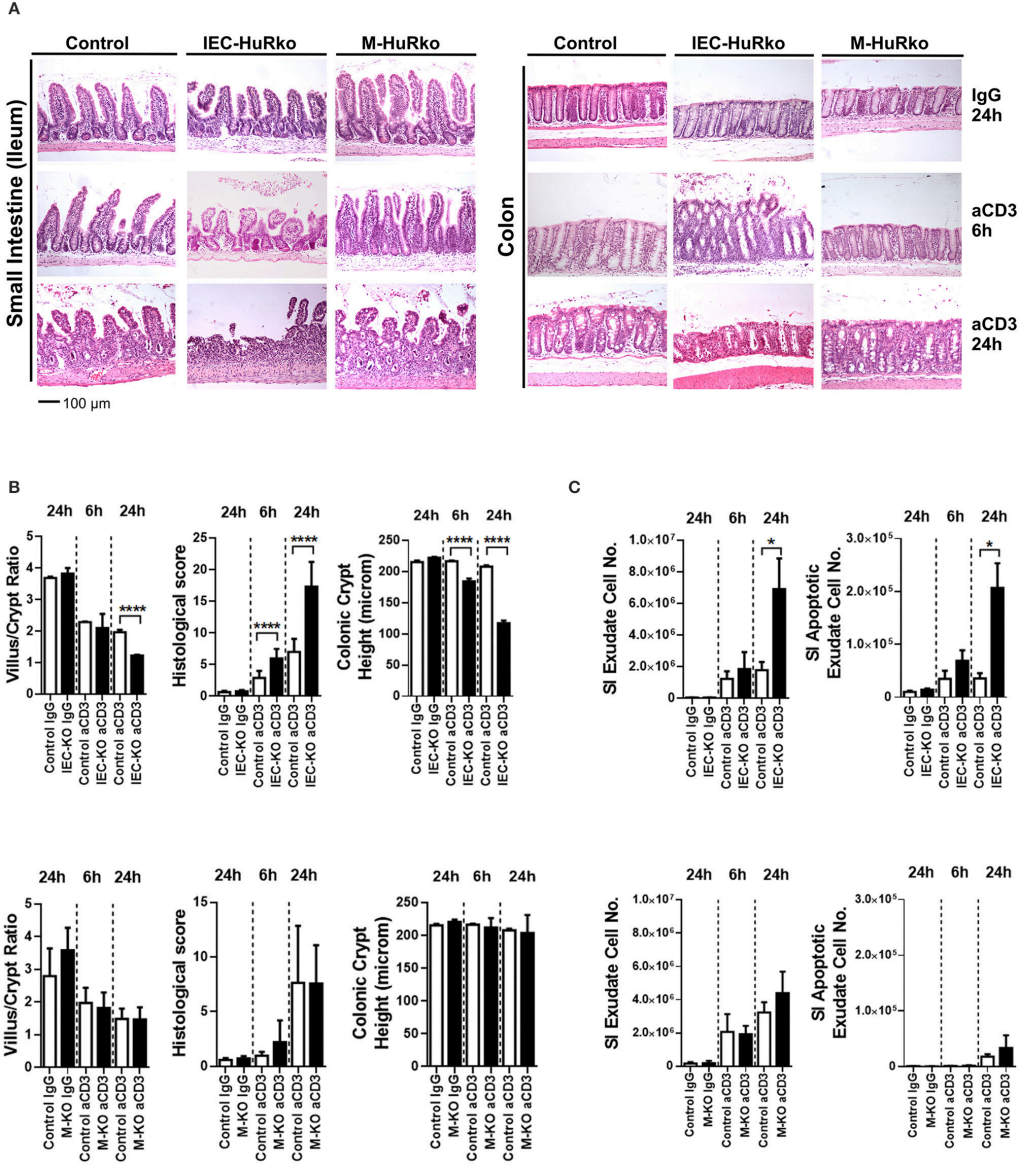
Loss of HuR in IECs –but not in myeloid cells- sensitizes the epithelium to acute inflammatory death signals. **(A)** Representative H&E histology of paraffin embedded intestines from control and HuR mutant mice exposed either to aCD3 or isotype IgG for the indicated times. **(B)** Quantification of villus/crypt length ratio, colonic crypt height and histological score of IEC-HuRko treated mice (upper panel) and M-HuRko mice (lower panel). *n* = 3–6/group/time point. Student unpaired *t-*test. **(C)** (left panels) Enumeration of cells expelled in lumen washes from the intestines of mice exposed to aCD3 or IgG control. (right panels) Flow cytometric determination of apoptotic cell numbers of the same exudates, analyzed 6 and 24 h post injections in IEC-HuRko (upper panel), M-HuRko (lower panel) mice and respective controls. *n* = 3–6/genotype/time point. Student unpaired *t*-test. All bar graphs show means (±SE). In all graphs, *,****, denote *p*-values < 0.05 and 0.0001 respectively.

Next, we assessed whether HuR is required to alter susceptibility to inflammatory damage either through enhanced pro-inflammatory innate signals or altered epithelial responses to such signals. Given the well-established role of HuR in proliferative regeneration of the intestine, we sought an acute model of inflammatory enteropathy that cannot be easily compensated by changes in regeneration. The systemic administration of agonistic antibodies targeting the CD3/T-cell receptor complex activates mucosal and submucosal T-cells to mount an aggressive local immune response that targets the intestinal epithelia for apoptosis via Fas, TNF, perforin and p53 signals (51). In control mice, disease is rapidly manifested in the small intestine and proceeds via the detachment of the apical epithelia, villus shortening, inflammation and crypt loss, within a period of 24 h, whereas the colon is only marginally affected (Figure 2A). IEC-HuRko mice were more susceptible to this model and displayed such symptoms even at 6 h post challenge whereas at 24 h they developed aggressive ulcerations (small intestine) and—surprisingly—apical degenerations in the large intestine (Figures 2A,B). Villus/crypt ratio and colonic crypt length were markedly reduced in IEC-HuRko mice at 24 h and at 6 h, respectively (Figure 2B). Enumeration of detached cells in lumen washes and annexin V/PI staining verified the increased shedding of apoptotic enterocytes in the intestines of IEC-HuRko mice (Figure 2C). On the other hand, the response of M-HuRko mice in this particular model was indistinguishable from that of the controls (Figures 2A–C). These data suggest that HuR in IECs exclusively desensitizes differentiated enterocytes to the death-promoting effects of inflammation thus revealing a first qualitative difference between IEC and myeloid HuR.

### Differential effects of myeloid vs. intestinal epithelial HuR in the control of *Citrobacter rodentium* induced colitis

To gain further insight into the possible role of intestinal and myeloid HuR in the elicitation of inflammation we employed a model of infectious colitis and pathogen control that also serves as a model of IBD observed during the invasion of enteropathic bacteria (52). *Citrobacter rodentium* is a natural pathogen of the intestinal mucosa that causes Transmissible Murine Colonic Hyperplasia and closely resembles the enteropathogenic and enterohaemorrhagic *Escherichia coli* strains (53). After oral administration, the bacterium colonizes the colon enforcing a series of interactive events in the resident tissue and the underlying immune compartments which—if failed—allow the infection to breach the barrier and become systemic. On the one hand, adhesion of the bacterium drives in part apical intestinal epithelial cell death to release infected cells, and this is counteracted by a rapid proliferative response that results in the lengthening of colonic crypts through propagation of transient amplifying cells (52). Underlying, this response, *C. rodentium* is initially phagocytosed by myeloid-derived immune compartments and recognized primarily via NLRP-3 inflammasome pathway leading to the release of pro-inflammatory interleukins. This cascade leads to a complex inflammatory response involving both innate and adaptive immune subsets, thus aiding the clearance of the pathogen and the production of antimicrobial proteins by colonocytes (52).

**Figure 3:**
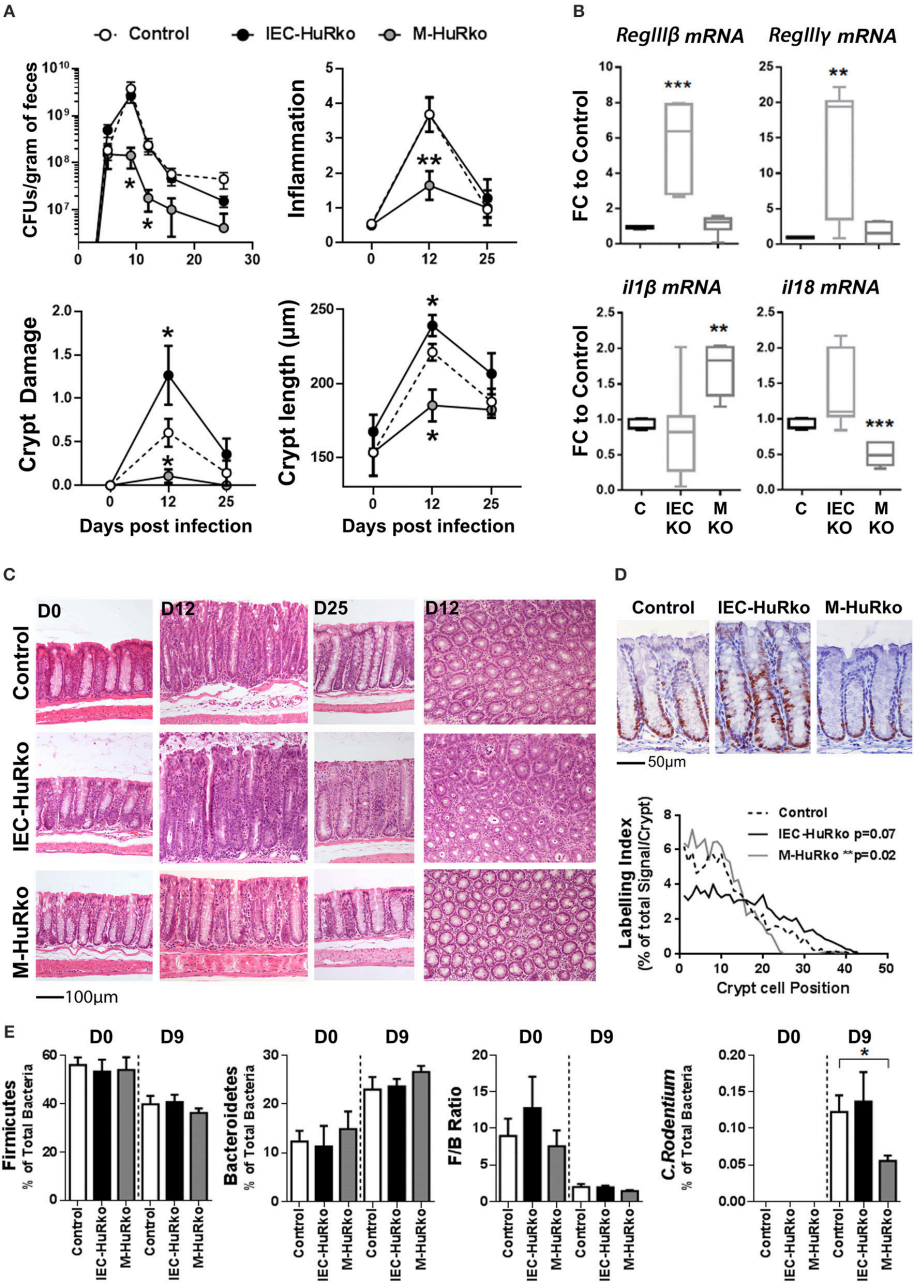
Differential susceptibility of IEC-HuRko and M-HuRko mice to *C. rodentium* infection. **(A)** (upper left) Fecal bacterial counts in mice orally infected with *C. rodentium*. (*n* = 16/group). Histological score of inflammation (upper right) and crypt damage (lower left) of uninfected (day 0) and infected mice (days 12 and 25). day 0, *n* = 5 per/group; day 12–16, *n* = 10–16 per/group. (Lower right) Crypt length measurements from H&E slides. Values are means (±SEM). **(B)** qPCR detection of mRNAs from distal colons on day 6 post infection expressed as fold change (FC) relative to control mice. *n* = 5–6 per/group. **(C)** Representative H&E histology of paraffin embedded distal colon sections of uninfected (day 0) and infected mice (day 12 and day 25 post infection). **(D)** Representative Ki67 immunohistochemical staining of distal colons from *C. rodentium* infected mice 12 days post infection (upper panel) and histograms of Ki67 labeling indices of proliferating epithelial cells in control and mutant mice measured from photomicrographs as in (upper panel). *n* = 6–7 mice/group. Wilcoxon Rank Sum test. **(E)** Relative abundance of Firmicutes, Bacteroidetes, *C. rodentium* taxa and Firmicutes to Bacteroidetes ratio (F/B Ratio) in the feces of uninfected (day 0) and infected (day 9) mice. Total intensity values for each taxon were used (see Supplementary Methods). Day 0, *n* = 7–14; day 9 *n* = 12–15. Student unpaired *t*-test. In all graphs *, **, *** denote *p*-values < 0.05, 0.01, and 0.001 respectively.

For our analyses, mutant and control mice were infected with *C. rodentium* and monitored via the estimation of the pathogen load in fecal matter for a period of 25 days; whereas histology was monitored at days 12 and 25 that correspond to peak disease and recovery phases, respectively. In control mice, the fecal bacterial load reached a plateau of 10^9^–10^10^ colony forming units (cfu) per gram of feces by day 9 post infection and dropped to a negligible minimum by day 25 (Figure 3A). The response correlated with detectable histological alterations in crypt damage, hyperplasia, inflammation, and goblet cell depletion during the peak phase of the disease, and also with the local synthesis of pro-inflammatory *il1*β and *il18* mRNAs and antimicrobial *RegIII*β and *RegIII*γ mRNAs (Figures 3A–C).

In IEC-HuRko mice, the kinetics of fecal bacterial loads was indistinguishable to that in controls, suggesting that these mice are capable of responding and clearing the infection (Figure 3A). However, and at the histological level, HuR-deficient epithelia displayed enhanced symptoms of degeneration, (e.g., crypt loss and apical epithelial detachment Figures 3A,C), relating to our findings in the T-cell mediated enteropathy model. Contrary however to the ascribed role of IEC-HuR in intestinal regeneration (16, 18), IEC-HuRko mice mounted a stronger crypt hyperplastic counter-response maintained even till day 25, which was associated with a near-significant upregulation in crypt proliferation as assessed via Ki67 immunostainings during the peak of the disease at day 12 (Figures 3A,C,D). Notably, the histological analysis of inflammatory infiltrates and the local synthesis of pro-inflammatory *il1*β and *il18* mRNAs did not reveal any significant changes (Figures 3A,B). However, HuR-deficient epithelia possessed higher levels of antimicrobial *RegIII*β and *RegIII*γ mRNAs (Figure 3B). Thus, the loss of HuR in IECs alters the resistance and antimicrobial properties of the epithelium to combat the infection independently of the underlying inflammatory process.

The response of the M-HuRko mice to *C. rodentium* was strikingly different. In these mice, bacterial loads did follow the proper kinetics but were substantially reduced and the bacterium was cleared more efficiently (Figure 3A). All histopathological hallmarks of the disease appeared significantly attenuated with a negligible effect in tissue damage and a near lack in the proliferative hyperplastic response of the infected crypts (Figures 3A,C,D). The clinical image of the infected M-HuRko mice correlated with an extensively heightened response in *il1*β – but not *il18*- mRNA suggestive of an increased activation of innate immunity (Figure 3B). Notably, the inducible synthesis of epithelial antimicrobial mRNAs in M-HuRko epithelia appeared similar to that in the infected controls. From these data collectively, we deduce that the loss of M-HuR aggravates the initial response of myeloid-derived immunity early in the process leading to the rapid elimination of *C. rodentium*. This suggests that similar to cases of pathologic inflammation, and in contrast to HuR in IECs, myeloid HuR acts to inhibit rather than promote beneficial inflammatory responses contributing in pathogen clearance.

The virulence and host resistance against *C. rodentium* may also depend upon the composition of the gut microbiota (52, 54); in turn *C. rodentium* itself is known to cause dysbiosis (55) with unpredictable effects in intestinal inflammation. Moreover, the pharmacologic inhibition of HuR has been shown to affect the composition of the intestinal microbiome (37). To examine whether the observed shifts in the susceptibility of our mutants could be accounted for by a shift in commensal microbiota we analyzed its composition in fecal matter derived from uninfected and infected mice using a proteomic approach. We restricted our analyses to the percentile representation of the bacterial phyla of Firmicutes and Becteroidetes since these are the most predominant in the mouse intestine (56–58), and correlated our measurements with the representation of *C. rodentium* itself since its colonizing properties alters the ratio of Firmicutes to Bacteroidetes (F/B ratio; Figure 3E). In our analysis we could not identify any changes in F/B ratio either in IEC-HuRko or in M-HuRko mice; this suggests that their cell-restricted deletion does not alter significantly the composition of the commensal microbiota. Changes in the presence of *C. rodentium* were further validated via our proteomics approach which verified its presence in IEC-HuRko mice and its reduced propagation in M-HuRko mice (Figure 3E). Still, the response of the commensal microbiota during the infection appeared indistinguishable to that in the controls (Figure 3E). Thus, the changes in the susceptibility to *C. rodentium* were not due to changes in the colonic microbiota but rather to cell intrinsic changes in IECs or myeloid cells, respectively.

### The combinatorial elevation of HuR discriminates the inflammatory from tumorigenic phases in CAC

**Figure 4:**
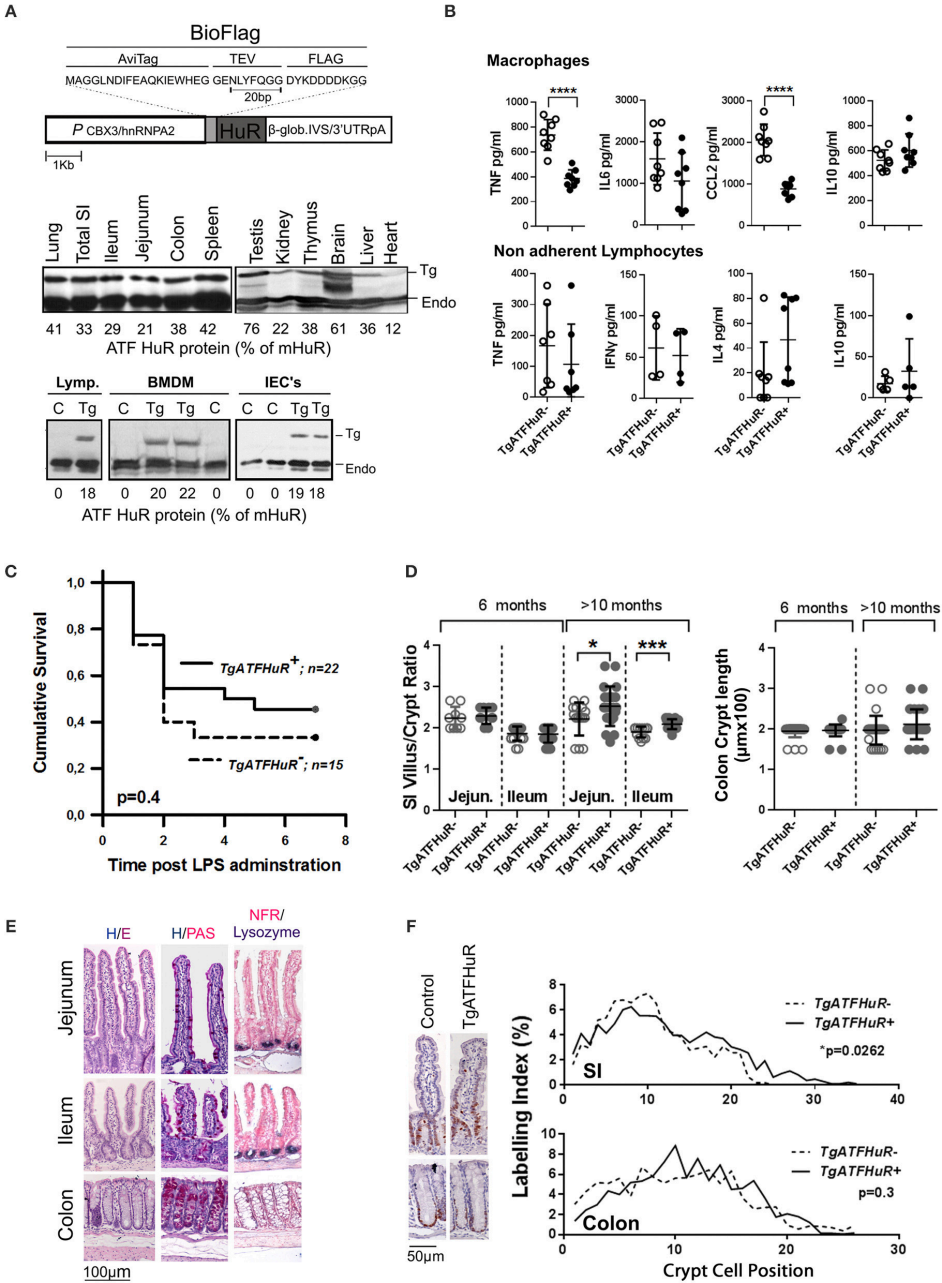
Generation and characterization of HuR overexpressing *TgATFHuR*^+^ mice. **(A)** (upper panel) Diagrammatic representation of the construct used for the generation of the TgATFHuR^+^ mice indicating the presence of: the CBX3/hnRNPA2 promoter; the tagged version of the human HuR with aminoacid composition of the Avidin and FLAG tags; the presence of β-globin intron/3′UTR that ensures a gene like structure and a 3′UTR and polyadenylation signal. (Middle panel) Representative immunoblots for the detection of the transgenic ATF-HuR protein in mouse tissues and quantitation to endogenous mouse HuR (mHuR). Membranes blotted with an anti-HuR antibody (3A2). (Bottom panel) Representative immunoblots for the detection of the transgenic ATF-HuR and endogenous mHuR proteins in extracts from *TgATFHuR*^+^ lymphocytes (lymp.), macrophages (BMDMs) and IECs. **(B)** ELISA detection of elicited inflammatory mediators in supernatants from cultured BMDMs (Upper panel), in the presence of Lipopolysaccharide (LPS; 100 ng/ml) for 24 h, and from non-adherent splenocytes (lymphocytes) in the presence of PMA/ionomycin. Data represent individual values/culture and mean (±SD). **(C)** Kaplan-Meier analysis following endotoxemia induction. Two experiments were performed with intraperitoneal administration of low (150 μg/25 g) and high (400 μg/25 g) doses of LPS, with similar results. Combined data from the two experiments are shown. Total group numbers are shown. *p*-value denotes result of Log-rank test. **(D)** Quantification of villus/crypt (jejunum and ileum) length ratio and colonic crypt height of younger (6 months old) and older (10–12 months old) mice. Data represent values of individual mice and mean (±SD). Student unpaired *t*-test. **(E)** Representative histology of sections from paraffin embedded intestinal tissue stained for general tissue architecture (hematoxylin/eosin), for Goblet cells (hematoxylin/periodic acid-Schiff), and for Paneth cells (anti-Lysozyme, blue stain, counterstained with Nuclear Fast Red). **(F)** (Left) Representative immunohistochemical staining of Ki67 (brown) counterstained with hematoxylin for detection of proliferating epithelial cells in resting TgATFHuR^−^ and TgATFHuR^+^ mice at the age of 2 months. (Right) Histograms of Ki67 labeling indices measured from photomicrographs as in (Left). Data compiled from *n* = 3–5 mice per group. *P*-values from Wilcoxon Rank Sum test. In all graphs *, ***, **** denote *p-*values < 0.05, 0.001, and 0.0001 respectively.

The data from the intestinal and myeloid HuR deficient mice indicate the differential requirement for HuR in controlling excessive inflammation from myeloid-derived immune cells, and in providing a balance between inflammatory damage of the epithelium and regenerative proliferation. Although informative, our data do not reveal what actually happens when HuR is elevated in conditions of IBD or cancer where myeloid and IEC functions co-exist. To gain such information we performed the opposite experiment by elevating HuR via the additional transgenic integration of an avidin/FLAG tagged-human HuR protein (*TgATFHuR*^+^ mice) driven by a promoter resistant to silencing (59) (Figure 4A). Five transgenic lines were originally generated; however only two expressed sufficient levels of HuR mRNA and from those we selected the one having the maximal expression pattern. In general, the expression of the transgene in this line (Tg6105; named hereafter as *TgATFHuR*^+^) was ubiquitous and yielded an increase in 30–40% in HuR protein across tissues (Figure 4A). At the cell subset level of lymphocytes, Bone Marrow Derived Macrophages (BMDMs) and IECs, the elevation was consistently around 20% (Figure 4A). Flow cytometric analysis of major immune cell subpopulations in bone marrow, thymus and spleen of *TgATFHuR*^+^ mice did not show any aberration in immune cell composition (Figure S3). Supernatants from activated macrophages and non-adherent splenocytes (lymphocytes) coming from *TgATFHuR*^+^ mice, revealed decreases in proinflammatory targets of HuR, TNF, and CCL2 in macrophages, as previously described for high expressing macrophage HuR transgenic lines (20), however effects upon other targets reported in other transgenic lines, were either not observed (like a reduction in IL6) or did not reach significance (like an increase in IL10) suggesting that the elevation of HuR is rather moderate (Figure 4B). This was also revealed when *TgATFHuR*^+^ mice were tested for sensitivity to systemic effects occurring in LPS-induced endotoxemia where these mice displayed only a mild trend of resistance to lethality (Figure 4C). With respect to intestinal epithelia, we noted a significant increase in the villus/crypt ratio in the small intestine of aged (>10 months) *TgATFHuR*^+^ mice, relating to an increase in crypt proliferation based on Ki67 staining but with no significant effects in cellularity (Figures 4D–F). We do note however that no changes were observed for colonic *TgATFHuR*^+^ crypts, which were indistinguishable from control crypts.

**Figure 5:**
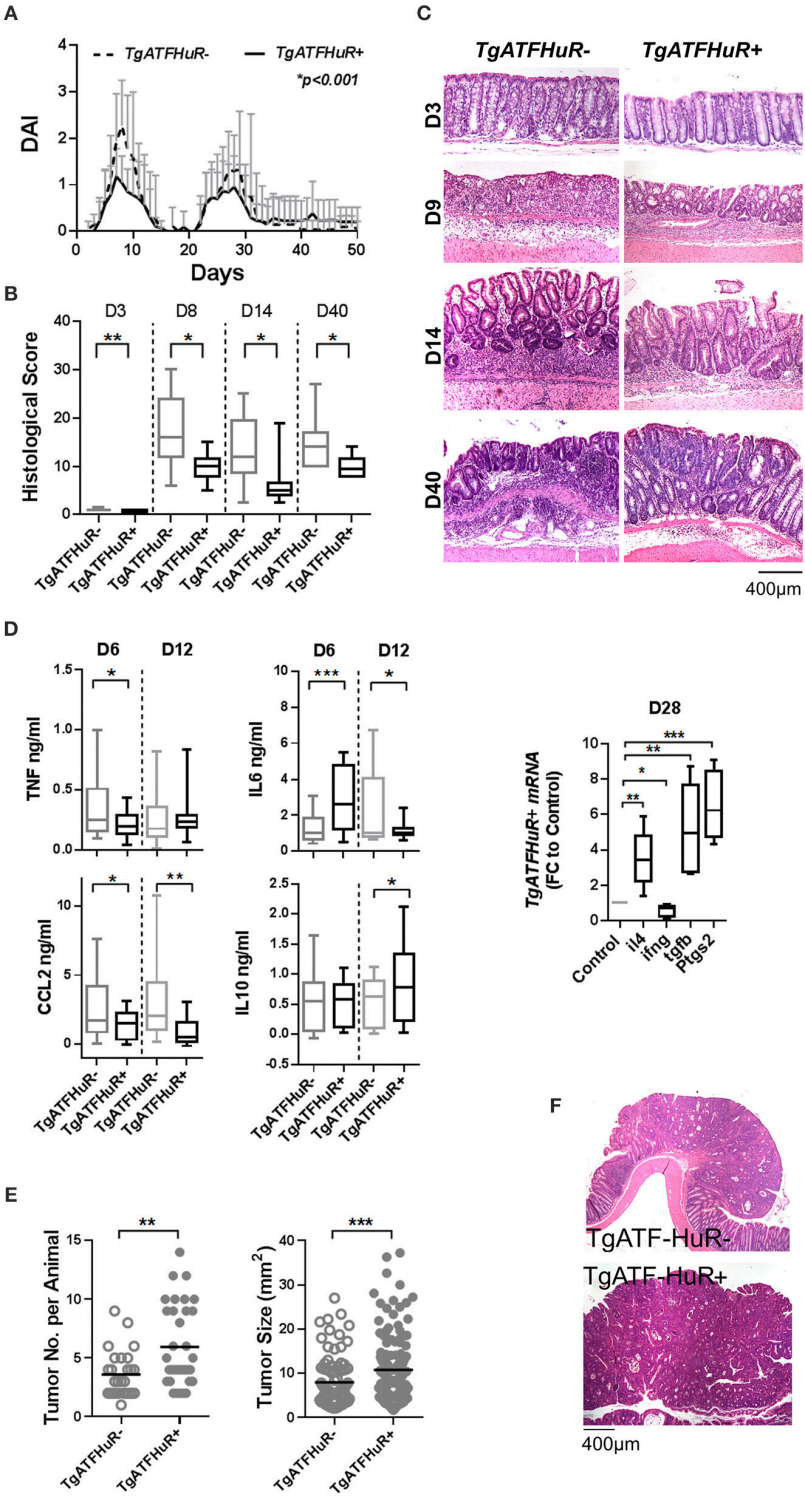
The combinatorial elevation of HuR attenuates chemically-induced colitis but augments CAC. **(A)** Macroscopic Disease Activity Index (DAI) during the 2 rounds of DSS administration (Days 0 and 20) in TgATFHuR^−^ and TgATFHuR^+^ mice. Line graphs depict mean values (±SD). *n* = 20–30/group. Kolmogorov-Smirnov test. **(B)** Histological scores of colitis at the indicated time points. Box plots show range and median. *n* = 8–12 mice/group. **(C)** Representative histology (H&E) of slides scored in **(B)**. **(D)** (Left and middle columns) ELISA quantification of inflammatory mediators in colon cultures isolated from DSS-treated mice at the indicated time points. Box plots show range and mean values. *n* = 3 cultures/group/time point. Student unpaired *t*-test. (Right) qPCR analysis of RNAs from DSS-treated colons of IEC-HuRko mice on day 28 after treatment expressed as fold change (FC) relative to control mice. *il4*, interleukin 4, *ifng*, interferon gamma, *tgfb*, transforming growth factor beta, *Ptgs2*, Prostaglandin-Endoperoxide Synthase 2. **(E)** Graphs depicting tumor number per mouse (left) and tumor size (right) in TgATF^−^ and TgATFHuR^+^ mice 60 days after challenge with DMH/DSS. Dots represent individual mice (left) and tumors (right); horizontal lines represent means. Student unpaired *t*-test. **(F)** Representative histology (H&E) of paraffin embedded colonic tumors measured in **(E)**. In all graphs, *, **, ***, denote *p*-value < 0.05, 0.01, and 0.001 respectively.

Collectively, *TgATFHuR*^+^ mice appear as a system that could reciprocate –in part- the functions of elevated HuR in the different subsets as they appear in cases of IBD and CAC (37). To test this, first we challenged *TgATFHuR*^−^ and *TgATFHuR*^+^ mice with the DMH/DSS protocol. During the inflammatory phases of the response, *TgATFHuR*^+^ mice displayed lower clinical inflammatory disease activity and histological scores than their controls and faster signs of epithelial restitution (Figures 5A–C). Analysis of the acute inflammatory profile of afflicted colons showed significant reductions in TNF and CCL2 production, an increase in anti-inflammatory IL10 and a variable response in IL-6 (Figure 5D), consistent with the partial loss of a pro-inflammatory character and the attenuated disease activity of *TgATFHuR*^+^ mice. This was also reflected at the chronic phase (day 28), were the mRNA of pro-inflammatory *ifn*γ was downregulated as opposed to transcripts of immunomodulatory *il4, ptgs2*, and *tgf*β that were upregulated in *TgATFHuR*^+^ mice (Figure 5D). In sharp contrast to their diminished pro-inflammatory response, *TgATFHuR*^+^ mice appeared more heavily susceptible to tumorigenesis, developing a significantly greater number of tumors of increased size per mouse (Figures 5E,F) which was different from what occurs when HuR is exclusively overexpressed in macrophages, where the reduction in inflammation correlated with a reduction in tumorigenesis (20). This suggests that during conditions of overexpression, the anti-inflammatory functions of HuR in immune cells segregate from its pro-tumorigenic effects in epithelia.

Finally, to assess whether the functions of TgATFHuR correlate with its cognitive characteristics we selected size-matched CAC-derived tumors from control and transgenic mice and performed Ribonucleoprotein Immunoprecipitation (RIP), with an anti-FLAG antibody, followed by Microarray Profile Analysis (Figure S4A,B). Approximately 2,000 genes were found bound by ATFHuR above control levels (Table S2). Gene Ontology enrichment analysis of these genes revealed groups involved in the processes of inflammation, cell cycle/apoptosis, development, epithelial function and others, consistent with previously published processes where HuR is involved (Figure S4C). Moreover, known target transcripts of HuR such as *Tnf*, *Ccr2, Cdc42* were also identified to be bound by ATFHuR in our RIP setting. Thus, the contrasting effects of ATFHuR relate to the true cognitive functions of HuR in the different cellular settings.

### The combinatorial elevation of HuR diversifies enterotoxic responses from inflammatory responses

**Figure 6:**
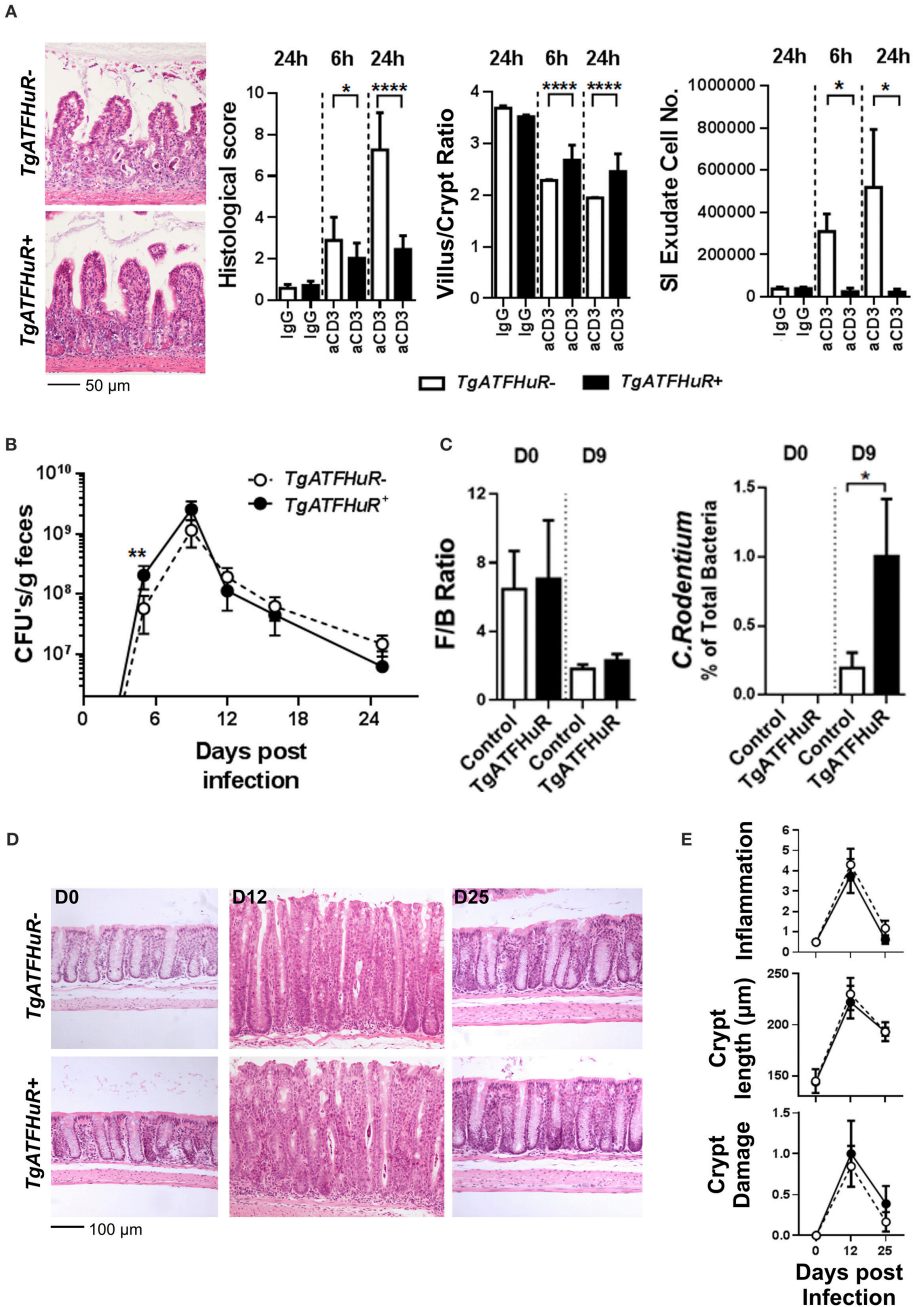
The combinatorial elevation of HuR protects the epithelium from acute inflammatory death signals but compromises inflammatory pathogen clearance. **(A)** Representative histology (H&E), histological scores, villus/crypt ratio quantification and determination of total cell number in the exudate of the small intestine at indicated time points post anti-CD3 antibody injections. Photomicrographs of ilea 24 h post injections are shown. Histological score and morphometry, *n* = 10/genotype/time point, Student unpaired *t*-test; Exudate cell number, *n* = 6–12/group, Mann-Whitney test. **(B)** Fecal bacterial counts in mice orally infected with *C. rodentium. n* = 10–25/group/time point. Student unpaired *t*-test. **(C)** Firmicutes to Bacteroidetes (F/B) ratio (left) and relative abundance of *C. rodentium* (right) in the feces of uninfected (day 0) and infected (day 9) mice. *n* = 8–12 mice/group/time point. Mann-Whitney test. **(D)** Representative histology of paraffin embedded distal colons from mice orally infected with *C. rodentium* at indicated time points post infection. **(E)** Histological parameters of *C. rodentium* colitis at the indicated time points post infection. Graphs show means (±SEM). *N* = 8–13/group/time point. In all graphs, *,**, ****, denote *p*-value < 0.05, 0.01, and 0.0001, respectively.

Next, we used a T-cell dependent model of inflammation to assess the role of overexpressed HuR in inflammatory progression vs. inflammatory epithelial damage following the same rationale as in the cell specific knockout mutants. First, the lack of a clear effect mediated by HuR in chronic inflammation was also conferred from our analyses of *TgATFHuR*^+^ *TNF*^Δ*ARE*/+^ mice that did not display any significant differences from their *TgATFHuR*^−^ *TNF*^Δ*ARE*/+^ controls (Figure S2). In sharp contrast, *TgATFHuR*^+^ mice were markedly more resistant to anti-CD3 mediated enterotoxicity with reduced symptoms of inflammatory degeneration and a near lack of a response in terms of exudate epithelial cells in the lumen (Figure 6A). As such, these mice displayed the opposite epithelial sensitivity of IEC-HuRko mice to inflammatory damage, which could connect also to their increased susceptibility to CAC. Finally, *TgATFHuR*^+^ mice displayed a significant delay in the early control of *C. rodentium* as indicated by fecal bacterial counts on the 9th day of the infection and the proteomic detection of representative peptides (Figures 6B,C). This indicated that the elevation of HuR compromised in part the efficiency of *C. rodentium* clearance. However, the remaining response was unaltered both in terms of the histological features as well as in the consistency of commensal microbiota (Figures 6B–E) suggesting a new balance in epithelial responses compensates to allow for the subsequent mechanism of defense against the bacterium.

By comparison, the response of *TgATFHuR*^+^ mice appears as exactly the opposite of what we could expect from the sum of differences in the debilitating mutations of HuR in myeloid-derived immune vs. epithelial compartments, thus verifying that HuR is acting as an anti-inflammatory regulator in myeloid-derived immunity and as a pro-survival and pro-tumorigenic factor in intestinal epithelia, with differential consequences in different contexts of intestinal inflammation.

## Discussion

In this study we followed a disease-oriented approach to examine whether HuR's independent functions in two distinct compartments co-existing in the intestinal mucosa (i.e., myeloid-derived immune cells and intestinal epithelial cells) contribute similarly or differentially in the same contexts of inflammatory intestinal disease. We chose to focus on the phenotypic effects of HuR deletion in these cell types, rather than on the molecular mechanisms that underlie them, in an effort to (a) highlight the specificity of HuR's functions thus providing a contextual roadmap for future molecular analyses; and (b) address the issue of HuR's exploitation in the clinical management of intestinal inflammation and degeneration. In doing so, we identified that the independent cellular responses guided by HuR's post-transcriptional functions have differential outcomes in intestinal inflammation.

In myeloid-derived immune compartments, HuR has primarily a regulatory role acting to maintain intestinal inflammation within physiological thresholds. This is supported by the differential susceptibility of our M-HuRko mice (augmented) to HuR overexpressing *TgATFHuR*^+^ mice (suppressed) in chemically-induced colitis; and is in line to previous observations in other settings of pathologic inflammation (17, 20, 38, 60). In molecular terms, this connects to HuR's capability of reducing the translation of several pro-inflammatory cytokine mRNAs (e.g., *Tnf, il6, Ccl2, Ccr2*) in innate immune cells, as opposed to their stability. Herein, we extend the regulatory functions of HuR toward beneficial myeloid-derived immunity functions required for host defense. This was exemplified by our data from the model of *C. rodentium* invasion where the loss of HuR enhanced pathogen clearance and limited inflammatory damage. This could connect to the capacity of HuR-deficient phagocytosing cells to uptake and clear the invading bacteria rapidly due to rapid CCL2/CCR2 mediated recruitment which seem to be connected to *C. rodentium* clearance (61) and whose synthesis is augmented in HuR-null macrophages supporting their enhanced homing at sites of inflammation (20). Alternatively, and though not addressed, HuR-null macrophages may display an enhanced inflammasome activity which is required for the clearance of this pathogen. This is indicated by several circumstantial evidence. In M-HuRko mice commensal bacteria respond properly to the invasion of *C. rodentium* suggesting that the bacterium can colonize the mucosa in these mice during the initial stages of the infection. On the other hand, the synthesis of antimicrobial RNAs known to be induced in IECs during the infection were not altered. The production of these RNAs in IECs is under the direct control of adaptive immune cells recruited as a result of the activation of myeloid-derived immune cells by the bacterium; as such, it signifies that adaptive immune response was not altered in M-HuRko mice despite their enhanced capability to control the infection. In contrast, we detected heightened levels of IL1b mRNA expression which could signify an enhanced activation of the inflammasome pathway that needs to be further examined at the molecular level.

The effects of myeloid HuR in the control of chronic inflammation appear less defined. In the TNF-mediated model of IBD, the loss of HuR did not alter the quality of the pathologic response-despite the signs for a delay in disease progression. This could be due to the complex hierarchy of pathologic responses supporting disease in this model which, besides myeloid-derived immune compartments, it is heavily dependent on the adaptive arm of immunity as well as on several stromal tissues (48–50). It is well-recognized that epithelial and stromal cells in the intestine express numerous receptors commonly found in immune cells and in this way they actively participate in the coordination of the immune response (62, 63). Alternatively, this could be due to the fact that TNF cannot be regulated by HuR since its mRNA is missing the HuR binding site. However, evidence that myeloid HuR can control chronic inflammation stem from the response of these mice during CAC. During CAC, a variety of toxic and regenerative inflammatory responses drive dysplasia and adenoma formation whereas subsequent tumor-associated inflammation (which includes Tumor Associated Macrophages) support tumor growth and invasion. The enhanced tumorigenesis and tumor growth observed in M-HuRko mice suggest that these responses may be either uncontrolled or qualitatively different.

The intestinal epithelial functions of HuR appear equally diverse. Our data are in line with previous suppositions on HuR acting positively toward intestinal epithelial programs of proliferation and regeneration. This is supported by the delayed restitution of DSS-challenged or irradiated epithelia when HuR is lost [this study and (18)] and its rapid occurrence when HuR is elevated; or by the diminishing effects of HuR's loss in tumor growth during CAC as opposed to its enhancing effect when HuR is overexpressed. Based on the extensive literature, this could be due to HuR's control over the Frizzled co-receptor Lrp6 (18), E-cadherin (36) and β-catenin (12) promoting Wnt-induced progenitor expansion; or the pleiotropic Rho GTPAse Cdc42 affecting proliferation, actin organization and migration (64). Although these mechanisms are now well-accepted, they reflect HuR's functions in stem and transient amplifying cells involved in regeneration. Our data indicate that HuR has a more predominant role in the homeostasis of differentiated enterocytes during inflammation for the preservation of intestinal barrier integrity under inflammatory stress via different means. This is heavily exemplified by the augmented response of IEC-HuRko mice in the model of T-cell mediated enterotoxicity which measures solely the response of enterocytes to inflammatory death signals. This connects to several post-transcriptional effects of HuR affecting directly or indirectly death responses. Directly, HuR may oppose enterocyte death, due to its positive regulation of prosurvival signals as in the cases of *Bcl2, Mcl1,ProT alpha, Sirt1*, and *PGC1a* as well as effectors of mitochondrial resistance (65, 66). Indirectly, this could also connect HuR's functioning in controlling the adherens junctions of enterocytes via its control over the stabilization of E-cadherin mRNA (36). To that end IEC-HuRko mice phenocopy those lacking E-cadherin, which display shedding and apoptosis of enterocytes, villus shortening and augmented colitis (67, 68). Conversely, *TgATFHuR*^+^ mice resemble mice with augmented E-cadherin/β-catenin junctions that are resistant to colitis (69, 70).

Arguably however, enterocyte cell death can be an inevitable side effect of a lack of continuous proliferation. However, our data on *C. rodentium* argue that in the physiological mucosa the prosurvival functions of HuR in enterocytes are more dominant than its effects in stem cell programs driving regeneration. In contrast to M-HuRko, IEC-HuRko mice possessed a normotypic response toward the clearance of the infection. However, HuR-deficient epithelia displayed a heightened level of tissue damage accompanied by a heightened proliferative response to compensate for that damage. Conversely, the elevation of HuR in *TgATFHuR*^+^ epithelia did not alter their hyperplastic response despite their resistance in enterotoxicity suggesting that the elevated HuR provided a new balance between inflammatory death and regeneration. Definitely however, and as indicated by the response of *TgATFHuR*^+^ mice to CAC, this balance is tilted toward the proliferative end providing benefits for the conversion of progenitors to dysplastic tissue, adenomas and carcinomas.

Our studies with *C. rodentium* suggest that HuR may also control the defensive properties of the epithelia. This is suggested by the augmented response in RNAs promoting antimicrobial defense (like RegIII) correlating with the proper resolution of the infection as opposed to the enhanced tissue damage. Whether this is directly regulated by HuR in IECs or via an indirect control over signals promoting antimicrobial gene expression in these cells remains to be determined.

Collectively, our data provide several indications for the clinical use of HuR inhibitors in combating intestinal inflammatory diseases. Several lead compounds have been reported in the literature. Their clinical use in intestinal inflammatory diseases is based on the assumption that they could act as selective and efficient means for inflammation control by blocking the activation of several pro-inflammatory, ARE-containing, cytokine mRNAs driving intestinal inflammation. In that sense they could provide more specific therapeutic means than generic immunosuppressants (e.g., corticosteroids, aminosalicylates, methotrexate, cyclosporine, azathioprine, and mercaptopurine) or more effective than single cytokine inhibitors (e.g., anti-TNF, anti-IL12/23 or NSAIDs for Cox-2) currently used to treat patients suffering from, for example, IBD.

Our findings indicate that these suppositions need to be reconsidered since HuR appears to have potent activities in regulating the acute responses of myeloid-derived mucosal subsets and the protection of the intestinal barrier from these responses. Similar considerations should be made for the applicability of HuR inhibitors in combating intestinal transformation and CRC. The main issue is the inflammatory context that either supports or impedes tumor initiation and progression. Our study provides a clear rationale as to why such inhibitors seem to fail in preclinical models of CAC since they could aggravate the early pro-inflammatory responses of myeloid-derived immune subsets driving IBD and enhance the genotoxic damage of the intestinal epithelial barrier thus enhancing the transformation process. We postulate that monotherapeutic schemes blocking HuR functions should be avoided in CRC arising in the context of IBD, where anti-cytokine therapies are also envisaged for applicability (e.g., anti-IL6).

Despite these issues, both our study and other studies on animal models support that HuR blockade could be of benefit in specific cases –or windows- of intestinal disease (37). For example, HuR inhibitors may be applicable in CRC's arising due to genetic mutations—like in the case of FAP and models of APC mutations- where inflammation may have a secondary role. In that context, HuR inhibition can clearly hinder the proliferative expansion of transformed cells, promote tumor death and aggravate an immune response targeting tumor cells for clearance. As such it could act as a more selective and efficient therapeutic strategy than current chemotherapeutics and surgical procedures for FAP. Perhaps, the anti-tumorigenic capabilities of HuR blockade could also be harnessed for CAC through the combinatorial-yet carefully controlled—use of anti-inflammatory drugs or drugs inhibiting the recruitment of myeloid-derived cells in the mucosa, alongside HuR inhibition regimes; or devise strategies for the selective uptake of HuR inhibitors by cancer cells thus bypassing the adverse targeting of myeloid-derived compartments. Finally, our data do support the beneficial use of HuR blockade in combating infections with enteropathogenic and enterohaemorrhagic bacteria since they could boost up beneficial immune and epithelial responses driving pathogen clearance. As such they could be used in combination with antibiotic or other antimicrobial regimes.

In general however, the ambiguity in the clinical effects of HuR blockade reflects the ambiguity in HuR functions both in cell intrinsic events and cellular interactions. As it stands, HuR drives both beneficial and adverse reactions in myeloid-derived and epithelial compartments. The molecular details for HuR functions in discriminating the post-transcriptional fate of tissue specific programs remain to be elucidated. To provide more definitive strategies on HuR blockade would require the dissection of HuR's protein and RNA partners that differentiate the beneficial from pathologic post-transcriptional programs and selectively target only the pathologic ones. To that end our study provides a framework to seek for such partnerships in the context of intestinal disease.

## Data availability statement

R-IP-Chip raw files that were generated for this study can be found on the Array-Express repository (https://www.ebi.ac.uk/arrayexpress/) with accession number E-MTAB-4018.

## Ethics statement

This study was carried out in accordance with the recommendations of Institutional Committee of Protocol Evaluation in conjunction with the Veterinary Service Management of the Hellenic Republic Prefecture of Attika and in accordance to national legislation and the European Union Directive 63/2010. Protocols were approved by Prefecture of Attika (licenses #5995/2012, 4371-4376/2014, #6198/2017, #3547/2018, #2824/2018).

## Author contributions

*In vivo* experiments were performed by EC-V, FI aided by MA; *TgATFHuR*^+^ mice generated by IK; histology and evaluation by AP (histopathologist), EC-V, FI, and MA; RIP experiments and analyses by GG, EC-V, and MR; microbiota and proteomic analyses by GS and MS; manuscript written by EC-V, FI, and DK.

### Conflict of interest statement

The authors declare that the research was conducted in the absence of any commercial or financial relationships that could be construed as a potential conflict of interest.
