# Supplementary material for: Divergent Innate and Epithelial Functions of the RNA-Binding Protein HuR in Intestinal Inflammation
Source: Front Immunol. 2018 Nov 23;9:2732. doi: 10.3389/fimmu.2018.02732 (PMC6265365; doi:10.3389/fimmu.2018.02732)
Supplement: Supplementary file 2 [file Data_Sheet_1.pdf]

frontiers

# Supplementary Information


- **Supplementary Figures and Tables (p. 3-8)**
- **Supplementary Methods (p. 9)**
- **Supplementary References (p. 10)**

*Supplementary Material*

# Divergent Innate and Epithelial Functions of the RNA-binding Protein HuR in Intestinal Inflammation

**Eleni Christodoulou-Vafeiadou[1#], Fotis Ioakeimidis[1#], Margarita Andreadou[1], Giorgos Giagkas[1], Yiorgos Stamatakis[1], Martin Reczko[1], Martina Samiotaki[1], Anastasios D. Papanastasiou[1], Ioannis Karakasiliotis[1], and Dimitris L. Kontoyiannis[1,2,&]**

[1]*Division of Immunology, Biomedical Sciences Research Center "Alexander Fleming", Vari, Greece;* [2]*Aristotle University of Thessaloniki, School of Biology, Department of Genetics, Development & Molecular Biology, Thessaloniki, Greece.*

#These authors contributed equally

[&]**Correspondence:** Dimitris L Kontoyiannis: kontoyiannis@fleming.gr

## 1 Supplementary Figures

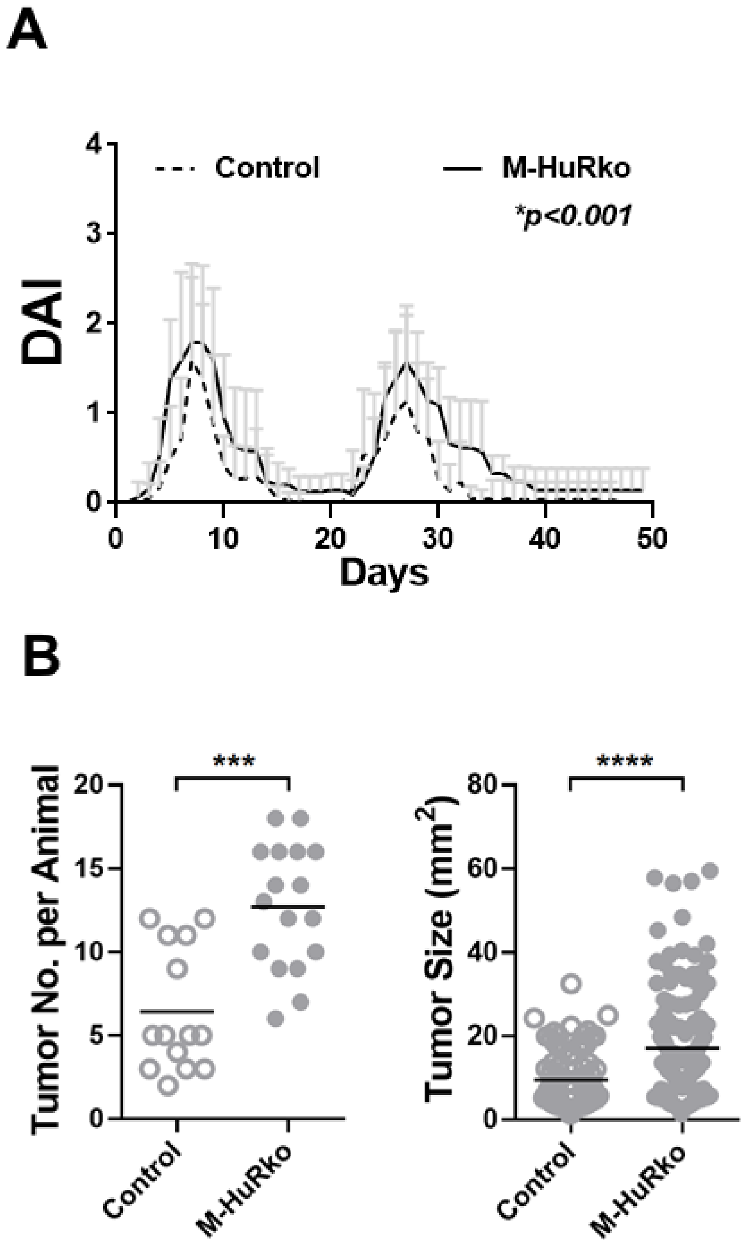


**Supplementary Figure S1. M-HuRko mice are more susceptible to DSS colitis and CAC. (A)** Macroscopic Disease Activity Index (DAI) during the 2 rounds of DSS administration (Days 0 and 20) in control and M-HuRko mice. Line graphs depict mean values (± SD). n=33/genotype. Kolmogorov-Smirnov test. **(B)** Tumor number per mouse and tumor size in control and M-HuRko mice 60 days after challenge with DMH/DSS. Dots represent individual mice and tumors and horizontal lines represent means. Student unpaired t test. ***, p<0.001, ****, p<0.0001. (Yiakouvaki et al., 2012) (1).

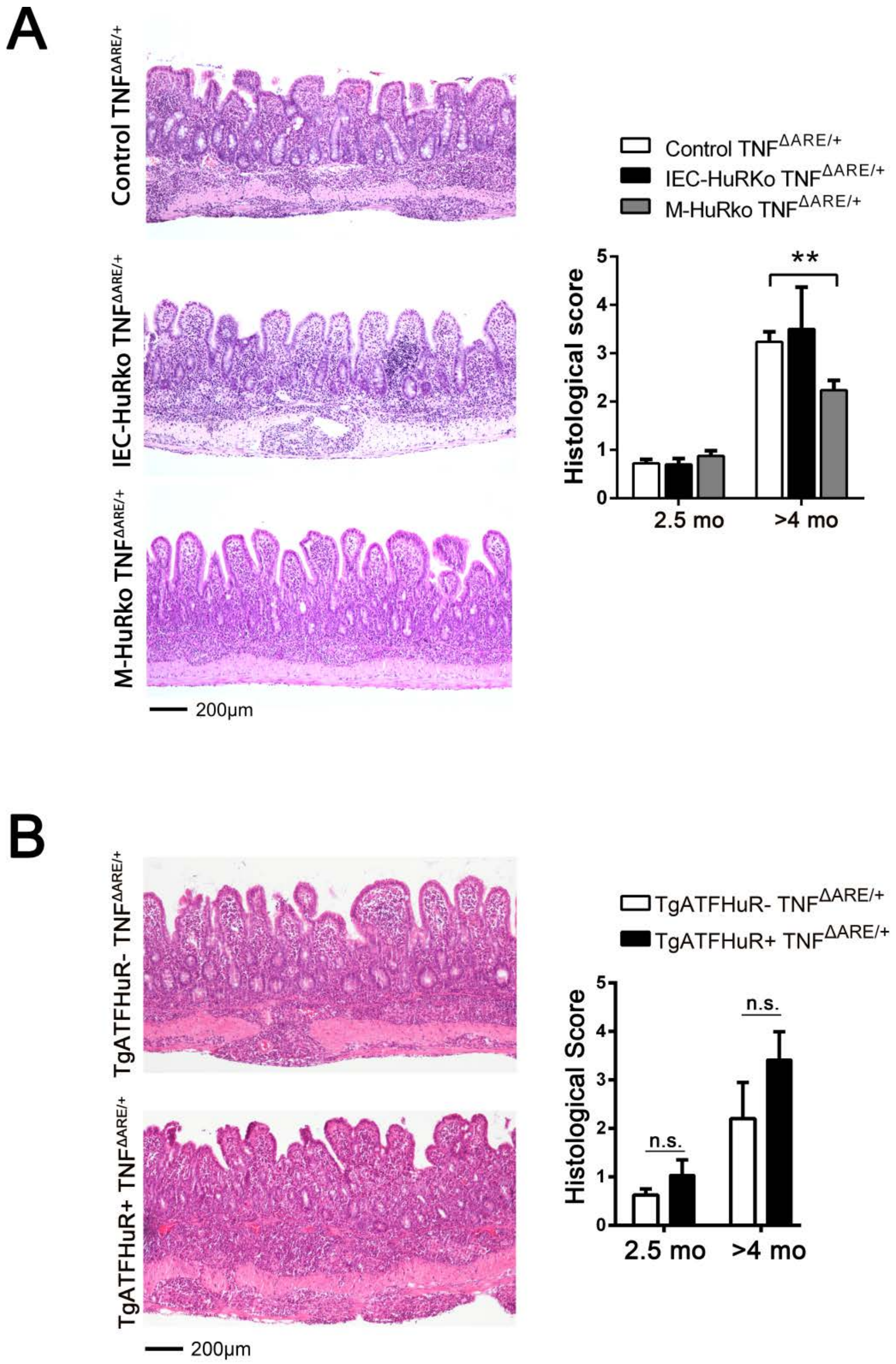


**Figure S2. Effect of HuR loss or increased expression on TNF-driven ileitis. (A,B)** Representative histology (H&E) and histological score of IEC-HuRko *TNF*$^{\Delta ARE/+}$, M-HuRko *TNF*$^{\Delta ARE/+}$ double mutants and Control *TNF*$^{\Delta ARE/+}$ mice (A) and of *TgATFHuR*$^{+}$ *TNF*$^{\Delta ARE/+}$ and *TgATFHuR*$^{-}$ *TNF*$^{\Delta ARE/+}$ mice (B). Paraffin embedded ileal sections from 5 month old mice are shown. Histological scores of 1-2.5 (2.5) and 4-6 (>4) month old mice were grouped together. Bar graph shows means (±SEM) of ileitis score. IEC-HuRko n=3-7/group/time point, M-HuRko n=9-19/group/time point. *TgATFHuR* n=3-8/group/time point. Mann-Whitney test. **, p<0.01.

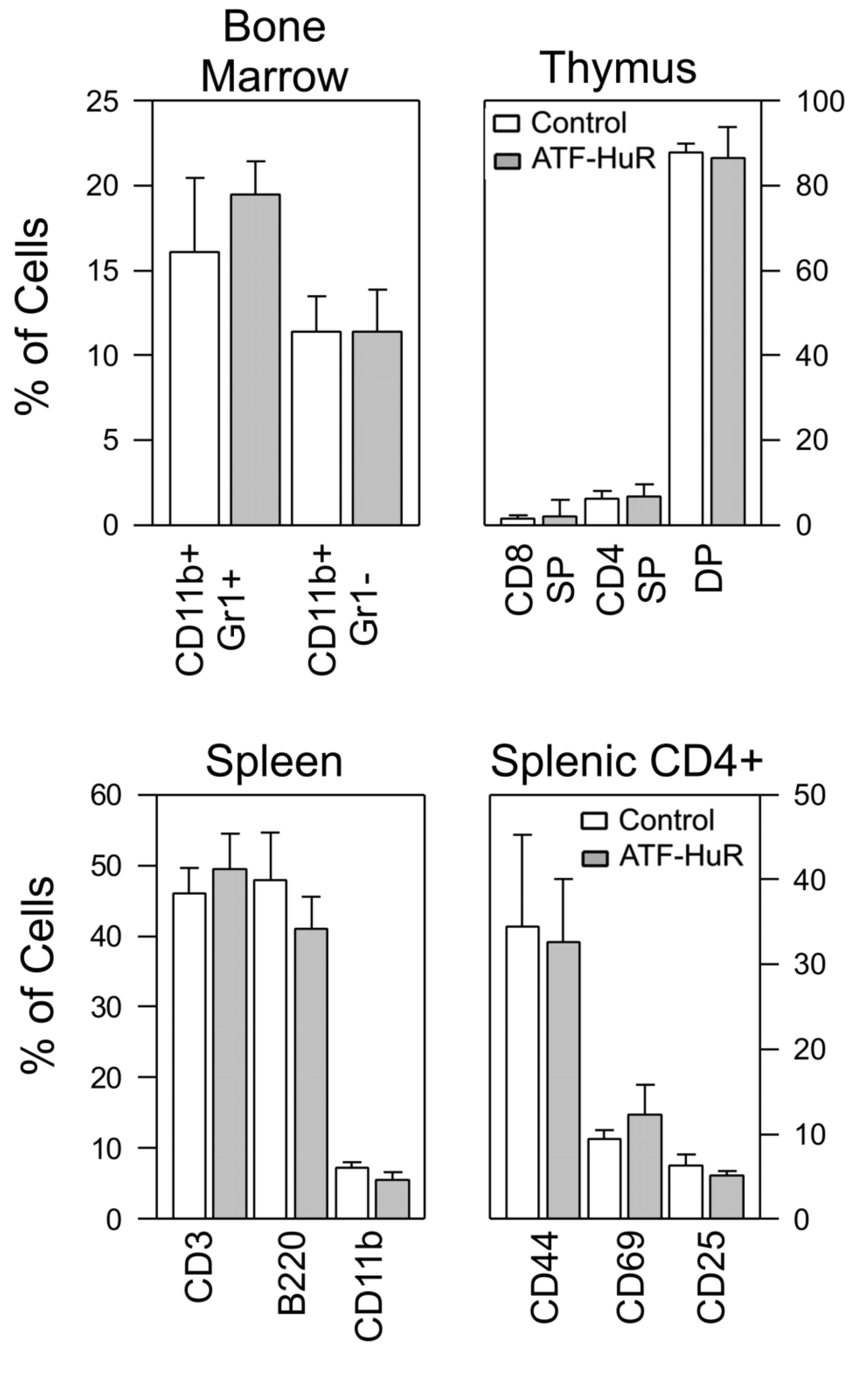


**Figure S3. Flow cytometric enumeration of major immune subtypes in control and *TgATFHuR+* mice show no major abnormalities**. Depicted are percentile counts of: macrophages ($CD11b^+/Gr1^-$) and polymorphonuclear cells ($CD11b^+/Gr1^+$) in bone marrow; $CD4^+$ (SP), CD8+(SP), and $CD4^+CD8^+$ (DP) T-lymphocytes in the thymus; mature T-lymphocytes (CD3), B-lymphocytes (B220) and myeloid (CD11b) cells in spleen; and activated/memory markers CD44, CD69 and CD25 on gated CD4+ splenic T-cells.

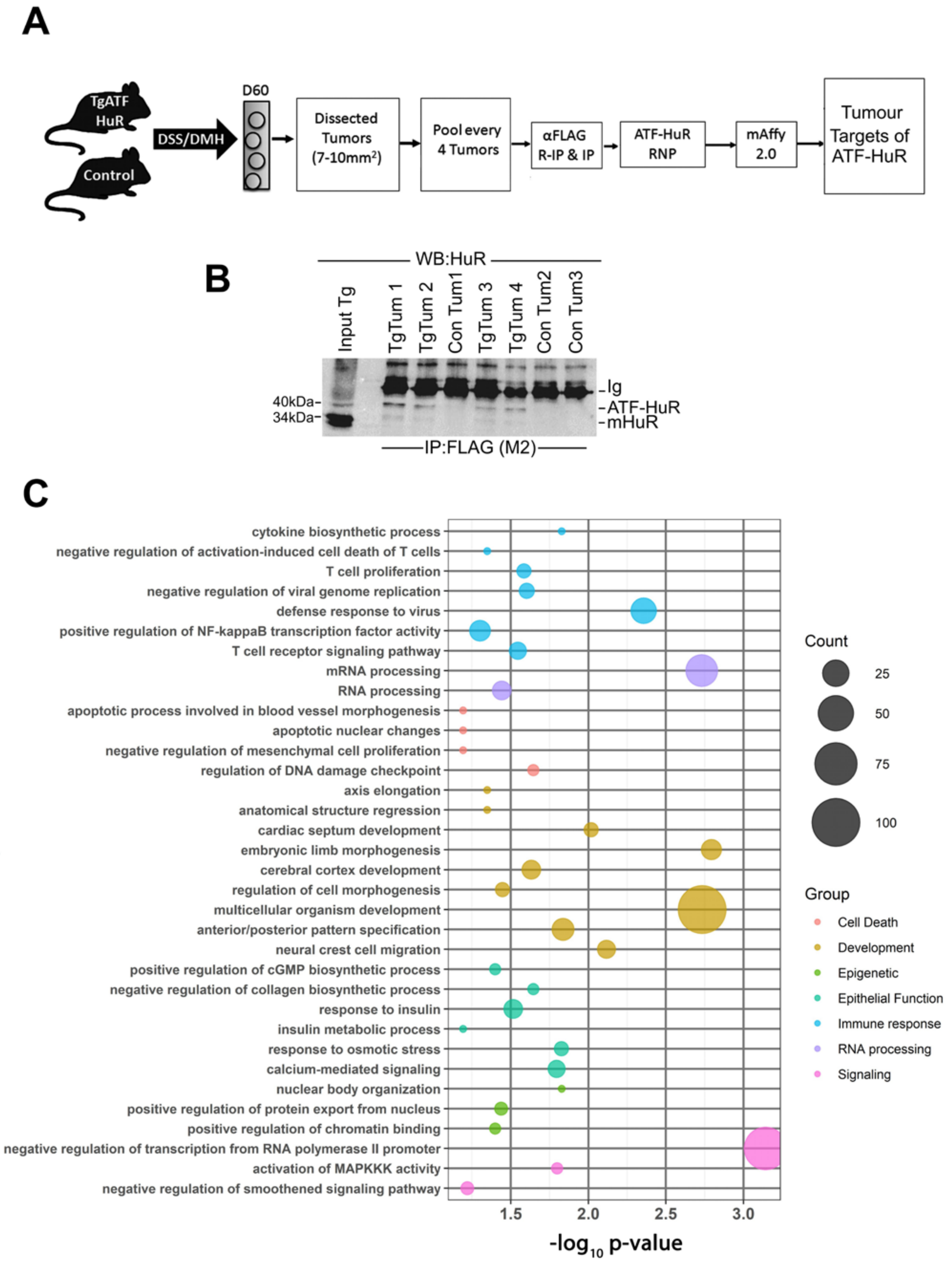
A
TgATF HuR
Control
DSS/DMH
D60
Dissected Tumors (7-10mm²)
Pool every 4 Tumors
αFLAG R-IP & IP
ATF-HuR RNP
mAffy 2.0
Tumour Targets of ATF-HuR
B
WB:HuR
Input Tg
TgTum 1
TgTum 2
Con Tum1
TgTum 3
TgTum 4
Con Tum2
Con Tum3
40kDa
34kDa
Ig
ATF-HuR
mHuR
IP:FLAG (M2)
C
cytokine biosynthetic process
negative regulation of activation-induced cell death of T cells
T cell proliferation
negative regulation of viral genome replication
defense response to virus
positive regulation of NF-kappaB transcription factor activity
T cell receptor signaling pathway
mRNA processing
RNA processing
apoptotic process involved in blood vessel morphogenesis
apoptotic nuclear changes
negative regulation of mesenchymal cell proliferation
regulation of DNA damage checkpoint
axis elongation
anatomical structure regression
cardiac septum development
embryonic limb morphogenesis
cerebral cortex development
regulation of cell morphogenesis
multicellular organism development
anterior/posterior pattern specification
neural crest cell migration
positive regulation of cGMP biosynthetic process
negative regulation of collagen biosynthetic process
response to insulin
insulin metabolic process
response to osmotic stress
calcium-mediated signaling
nuclear body organization
positive regulation of protein export from nucleus
positive regulation of chromatin binding
negative regulation of transcription from RNA polymerase II promoter
activation of MAPKKK activity
negative regulation of smoothened signaling pathway
1.5
2.0
2.5
3.0
-log10 p-value
Count
25
50
75
100
Group
Cell Death
Development
Epigenetic
Epithelial Function
Immune response
RNA processing
Signaling

**Figure S4**. **Identification of ATFHuR binding targets in colonic tumors. (A)** Strategy for the identification of ATFHuR binding targets in DMH/DSS induced tumors. **(B)** Immunodetection of HuR and ATFHuR in anti-FLAG R-IP assays from colon tumors collected as described in (A) from *TgATFHuR*$^{+}$ (TgTum) and *TgATFHuR*$^{-}$ (Con Tum) mice. Detection using anti-HuR antisera (3A2). RNA from these RNPs was used for microarray hybridizations. **(C)** Gene Ontology enrichment of genes bound by ATFHuR. Bubble plot shows enriched terms and their corresponding p-value for genes bound by ATFHuR. Bubble size indicates the number of genes (total n=1898) related to each biological term provided by DAVID. Terms with a p-value<0.065 are shown and were grouped based on relevant biological function. Details are provided in **Supplementary Methods**.

## 1.1 Supplementary Table S1. Primers used for qPCR analyses

| Name | Sequence (5’→ 3’ orientation) |
|---|---|
| Il-4 Forward | GGTCTCAACCCCCAGCTAGT |
| Il-4 Reverse | GCCGATGATCTCTCTCAAGTGAT |
| Il-18 Forward | GCCTCAAACCTTCCAAATCA |
| Il-18 Reverse | TGGATCCATTTCCTCAAAGG |
| Il-1β Forward | GCAACTGTTCCTGAACTCAACT |
| Il-1β Reverse | ATCTTTTGGGGTCCGTCAACT |
| Ifng Forward | GAGGTCAACAACCCACAGGT |
| Ifng Reverse | GGGACAATCTCTTCCCCACC |
| B2M Forward | TTCTGGTGCTTGTCTCACTGA |
| B2M Reverse | CAGTATGTTCGGCTTCCCATTC |
| Tgfb1 Forward | TGACGTCACTGGAGTTGTACGG |
| Tgfb1 Reverse | GGTTCATGTCATGGATGGTGC |
| Ptgs2 Forward | TCAGTTTTTCAAGACAGATC |
| Ptgs2 Reverse | TCTCTACCTGAGTGTCTTTG |
| RegIIIβ Forward (2) | ATGGCTCCTACTGCTATGCC |
| RegIIIβ Reverse (2) | GTGTCCTCCAGGCCTCTTT |
| RegIIIγ Forward (2) | ATGGCTCCTATTGCTATGCC |
| RegIIIγ Reverse (2) | GATGTCCTGAGGGCCTCTT |

## Supplementary Methods

### Microbiome analysis

Data Processing Protocol: The raw files were analyzed using a two-step strategy similar as in published metaproteomic analyses (3, 4). The raw files were searched with the XTandem engine (Galaxy version 1.1.1) against the merged fasta file that was generated from: i) the gut proteome of mice (generated from NGS analysis of fecal samples from 184 different mice) (5), ii) Uniprot all and iii) the Uniprot *C. rodentium* proteome. The search conditions were 10 ppm for MS1 and 0.5 Daltons mass tolerance for MS/MS. The protein lists generated were merged and filtered for redundant entries creating a new FASTA file containing 678,379 entries.

The Proteome Discoverer searches using the Percolator algorithm generated FDR filtered results with a quality score (q-value). The percolated files were used to build a chromatographic library using Skyline (version 4.1) with 0.99 as a q-value cut-off score. The chromatograms were imported as centroided masses with 10 ppm threshold and a range of 5 min around the retention time of the corresponding peptide in the library. The data were filtered to remove proteins with one peptide and the remaining peptides were finally filtered based on their uniqueness at taxonomic phylum level using the Unipept metaproteomic-biodiversity analysis (6). Totally, thousands of bacterial-specific, phylum-specific and *C. rodentium* specific peptides with intensity values were summed in order to generate the biomass of total bacteria and of the respective bacterial phyla present in the mouse fecal samples. These values were normalized to the weighed fecal mass for each sample.

### Microarray (Chip) Profile Analysis

300 ng of total RNA were used to generate biotinylated complementary RNAs, (cRNA) which were hybridized onto Affymetrix Mouse Gene 2.0 ST in accordance to the protocols of the Genomics Unit of BSRC “Alexander Fleming”. (http://www.fleming.gr/facilities/genomics). Analysis and normalization was performed using oligo package (version 1.42.0) in R (7). Significance in differential gene expression was determined by the probability of positive log ratio (PPLR score) for each probe set using puma package (8). Genes were considered bound by ATF-HuR for probe sets with positive expression change between *TgATFHuR*$^{+}$ and *TgATFHuR*$^{-}$ (n=3 per genotype). Significance of binding was defined as 1.1-fold expression change and PPLR score above 0,8. Complete list of bound genes that meet these criteria of statistical significance are provided in supplementary .xls file. Pathway and gene ontology (GO) enrichment analyses were carried out on bound genes using the Database for Annotation, Visualization, and Integrated Discovery (DAVID; http://david.abcc.ncifcrf.gov/ home.jsp). Biological process terms were visualized in bubble-plot generated in ggplot2 in R (9).

### Flow cytometry of immune cells

Immune cells were isolated through standard procedures, counted, stained with fluorescently labeled antibodies and analyzed with a FACS Canto II flow cytometer (BD Biosciences). Acquired data were analyzed using FlowJo (V 7.2.5; Tree Star). Antibodies used: CD4-Alexa700(GK1.5), CD44-APC(IM7) and CD69-PE-Cy7(H1.2F3) from eBioscience; CD8a-APC-Cy7(53-6.7) and Gr1/Ly-6G from BD Biosciences, CD3e-APC-Cy7(145-2C11), CD25-PE(PC61) and CD45R/B220-APC-C7(RA3-6B2) from Biolegend; CD11b-Alexa647(M1/70) from BDPharmingen.

**Supplementary References**

1. Yiakouvaki A, Dimitriou M, Karakasiliotis I, Eftychi C, Theocharis S, Kontoyiannis DL. Myeloid cell expression of the RNA-binding protein HuR protects mice from pathologic inflammation and colorectal carcinogenesis. *J Clin Invest* (2012) 122(1):48-61. Epub 2011/12/29. doi: 10.1172/JCI45021

45021 [pii]. PubMed PMID: 22201685; PubMed Central PMCID: PMC3248801.

2. Zheng Y, Valdez PA, Danilenko DM, Hu Y, Sa SM, Gong Q, et al. Interleukin-22 mediates early host defense against attaching and effacing bacterial pathogens. *Nat Med* (2008) 14(3):282-9. Epub 2008/02/12. doi: 10.1038/nm1720

nm1720 [pii]. PubMed PMID: 18264109.

3. Kleiner M, Thorson E, Sharp CE, Dong X, Liu D, Li C, et al. Assessing species biomass contributions in microbial communities via metaproteomics. *Nat Commun* (2017) 8(1):1558. Epub 2017/11/18. doi: 10.1038/s41467-017-01544-x

10.1038/s41467-017-01544-x [pii]. PubMed PMID: 29146960; PubMed Central PMCID: PMC5691128.

4. Blank C, Easterly C, Gruening B, Johnson J, Kolmeder CA, Kumar P, et al. Disseminating Metaproteomic Informatics Capabilities and Knowledge Using the Galaxy-P Framework. *Proteomes* (2018) 6(1). Epub 2018/02/01. doi: E7 [pii]

10.3390/proteomes6010007

proteomes6010007 [pii]. PubMed PMID: 29385081; PubMed Central PMCID: PMC5874766.

5. Xiao L, Feng Q, Liang S, Sonne SB, Xia Z, Qiu X, et al. A catalog of the mouse gut metagenome. *Nat Biotechnol* (2015) 33(10):1103-8. Epub 2015/09/29. doi: 10.1038/nbt.3353

nbt.3353 [pii]. PubMed PMID: 26414350.

6. Mesuere B, Debyser G, Aerts M, Devreese B, Vandamme P, Dawyndt P. The Unipept metaproteomics analysis pipeline. *Proteomics* (2015) 15(8):1437-42. Epub 2014/12/06. doi: 10.1002/pmic.201400361. PubMed PMID: 25477242.

7. Carvalho BS, Irizarry RA. A framework for oligonucleotide microarray preprocessing. *Bioinformatics* (2010) 26(19):2363-7. Epub 2010/08/07. doi: 10.1093/bioinformatics/btq431

btq431 [pii]. PubMed PMID: 20688976; PubMed Central PMCID: PMC2944196.

8. Liu X, Milo M, Lawrence ND, Rattray M. Probe-level measurement error improves accuracy in detecting differential gene expression. *Bioinformatics* (2006) 22(17):2107-13. Epub 2006/07/06. doi: btl361 [pii]

10.1093/bioinformatics/btl361. PubMed PMID: 16820429.

9. Wickham H, Grolemund G. *R for data science : import, tidy, transform, visualize, and model data*. First edition. ed. xxv, 492 pages p.
